# TCP19 regulates nitrogen‐dependent sheath blight susceptibility by modulating nitrogen uptake and signalling in rice

**DOI:** 10.1111/pbi.70224

**Published:** 2025-06-26

**Authors:** Tiange Zhou, Huan Chen, Xu Jiang, Hongyao Zhu, Qiujun Lin, Ki‐Hong Jung, Xiaofeng Zhu, Yuan Hu Xuan

**Affiliations:** ^1^ State Key Laboratory of Elemento‐Organic Chemistry and Department of Plant Protection, National Pesticide Engineering Research Center, College of Chemistry Nankai University Tianjin China; ^2^ College of Plant Protection Shenyang Agricultural University Shenyang China; ^3^ Key Laboratory of Saline‐Alkali Vegetation Ecology Restoration Ministry of Education (Northeast Forestry University) Harbin China; ^4^ Graduate School of Green‐Bio Science and Crop Biotech Institute Kyung Hee University Yongin Korea; ^5^ Institute of Agricultural Quality Standards and Testing Technology Liaoning Academy of Agricultural Sciences Shenyang China

**Keywords:** nitrogen, TCP19, PIL15, IDD10, N signalling, rice

## Abstract

The excessive application of nitrogen fertilization improves rice yield, but increases disease severity. However, the underlying molecular mechanisms remain unclear. Here, we conducted a comparative analysis of nitrogen‐ and *R. solani*‐regulated transcriptomes and identified *TEOSINTE BRANCHED, CYC, PCF 19* (*TCP19*), that was a key regulator of nitrogen use efficiency (NUE), as a potential link between nitrogen metabolism and sheath blight (ShB) regulation. Inoculation of *tcp19* and *TCP19 OXs* with *R. solani* revealed that *TCP19* negatively regulated ShB resistance independent of nitrogen conditions. TCP19 expression was suppressed under moderate nitrogen (MN) but induced under high nitrogen (HN) conditions. Furthermore, TCP19 directly activated *Dense and Erect Panicle 1* (*DEP1*) while repressing *nitrate transporter 1.1B* (*NRT1.1B*), *ammonium transporter 1;2* (*AMT1;2*) and *pathogenesis‐related 1b* (*PR1b*). Notably, TCP19 induced by HN conditions further strengthened this regulation. Phytochrome interacting factor like protein 15 (PIL15), a TCP19 interactor, directly activated *DEP1* and *AMT1;2* while repressing *NRT1.1B*. Additionally, the key nitrogen signalling regulator Indeterminate domain 10 (IDD10) interacted with both TCP19 and PIL15 and inhibited *DEP1* activation by TCP19 and PIL15. Interestingly, DEP1 competitively interacted with IDD10 to release TCP19 and PIL15. Overall, our findings elucidate the mechanisms by which TCP19 regulates nitrogen signalling in rice ShB resistance, highlighting TCP19‐*PR1b* signal under HN conditions as a key factor contributing to increased disease severity.

## Introduction

Rice (*Oryza sativa*) is a staple food for over half of the global population, with an increasing demand for its cultivation (Fairhurst and Dobermann, 2002). However, rice sheath blight (ShB) caused by the fungal pathogen *Rhizoctonia solani* Kühn poses a significant threat to global rice production (Singh *et al*., 2019), which has been regarded as one of the most destructive diseases affecting rice production, particularly in warm and humid regions (Eizenga *et al*., 2002; Groth and Bond, 2007). Management strategies for ShB include the development of resistant cultivars, crop rotation and fungicide applications (Kumar *et al*., 2009; Lee, 1983; Park, 2008). In contrast, pathogen adaptability and resistance to fungicides continue to challenge effective control (Mew *et al*., 2004). Although numerous resistance genes and quantitative trait loci (QTLs) have been identified, providing valuable targets for breeding programmes (Yadav *et al*., 2015), the molecular mechanisms underlying disease regulation remain unclear. Omics studies have revealed complex signalling pathways and defence responses to pathogen infection (Karasov *et al*., 2014; Khodayri *et al*., 2009; Peng Yuan *et al*., 2020; Wang *et al*., 2021; Yadav *et al*., 2015; Yang *et al*., 2022). Significant research gaps remain, hindering a comprehensive understanding of disease dynamics and signalling during infection; further research is needed to establish direct molecular connections.

Nitrogen (N) is essential for rice growth, development and yield improvement, and increased N fertilizer input significantly contributes to rice production (Makino, 2011). Consequently, N fertilization has become a critical practice in rice cultivation (Peng *et al*., 2010). However, the escalating application of N fertilizers has not only reduced nitrogen use efficiency (NUE) but also increased the susceptibility of rice to disease such as ShB (Fei and Peng, 2017; Peng *et al*., 2002), prompting researchers to focus on improving NUE and resistance. Several genes associated with NUE have been identified, including *TEOSINTE BRANCHED, CYCLOIDEA, PCF 19* (*TCP19*), *nitrate transporter 1.1B* (*NRT1.1B*), *ammonium transporter 1* (*AMT1*), and *Dense and Erect Panicle 1* (*DEP1*). These genes play crucial roles in nitrogen uptake and signalling, while also influencing plant defence responses. TCP19 has been shown to regulate tillering response to nitrogen (TRN) and NUE. Field tests of a near‐isogenic line (NIL) carrying *TCP19*‐H (NIL^*TCP19*‐H^) demonstrated more tillers, grain yields and higher NUE under low and moderate nitrogen conditions (Liu *et al*., 2021b). Further research indicates that GATA8 directly binds to the downstream genes *TCP19* and *AMT3.2* to regulate ammonium uptake and facilitate efficient tillering in rice to increase NUE (Wu *et al*., 2024). Additionally, the *DEP1* gene has been identified as a major QTL for NUE, with the rice genome carrying the dominant *dep1‐1* allele exhibiting lower nitrogen sensitivity and improved nitrogen uptake and assimilation, resulting in greater NUE and yield (Huang *et al*., 2009, 2022; Sun *et al*., 2014; Zhao *et al*., 2019). Plants can absorb nitrogen from the soil primarily in the form of nitrates and ammonium, with ammonium being the major form in paddy fields and the primary source of nitrogen for rice (Li *et al*., 2017). AMTs play a crucial role in nitrogen uptake in rice plants. *AMTs* in rice have been extensively investigated for their expression patterns, localization and responses to ammonium (Li *et al*., 2009). The yield assessments of *AMT* knockout mutants have suggested that single or double knockouts of *AMT1* do not significantly affect the yield, whereas triple knockouts severely impair plant growth and yield (Konishi and Ma, 2021). Nitrate is the main form of N in aerobic fields, and primary nitrate transporters (NRT/PTR/NPF) have been identified in rice (Feng *et al*., 2011; Li *et al*., 2017). *NRT1*, the first identified nitrate transporter in rice, is primarily expressed in the roots (Lin *et al*., 2000), and *NRT1.1B* has the potential to improve NUE (Hu *et al*., 2015; Zhang *et al*., 2019). Additionally, allelic variation in *NR2*, which encodes a nitrate reductase, underpins the differences in nitrate assimilation and NUE between indica and japonica rice, providing a key target for improving japonica yield (Gao *et al*., 2019). Together, these findings highlight the complex interplay between NUE and disease resistance, necessitating further exploration of its function in the NUE‐ShB interaction.

Excessive N fertilizer application not only reduces rice NUE but also exacerbates the incidence of diseases such as ShB (Savary *et al*., 1995). The severity of ShB is closely linked to N application, and excessive fertilization increases the occurrence of diseases (Fei and Shao‐bing, 2017; Li *et al*., 2012; Tang *et al*., 2007). Although NUE is recognized as an indicator of ShB resistance, the specific signalling mechanisms connecting N and ShB resistance remain unclear. N uptake and assimilation pathways play a key role in shaping plant immunity. Previous studies have indicated that *AMT1* can positively influence ShB resistance by regulating ammonium uptake and assimilation (Wu *et al*., 2022). The susceptibility of *NRT1.1B* to ShB can be affected by the N source in the environment, with the *nrt1.1b* mutant being susceptible to nitrate conditions but more resistant when ammonium is the sole N source (Li *et al*., 2023). In addition to N transporters, other transcription factors regulate N‐related genes and responses. For instance, our previous study suggested that indeterminate domain 10 (IDD10) could directly activate genes involved in ammonium uptake and N assimilation (Xuan *et al*., 2013), while negatively regulating rice ShB resistance (Jung *et al*., 2023). TCP19 is known to induce TRN and NUE in rice by negatively regulating DWARF AND LOW‐TILLERING (*DLT*) (Liu *et al*., 2021b), but its role in disease resistance remains unexplored.

In this study, we identified TCP19 as a negative regulator of ShB resistance. TCP19 directly control the expression of *AMT1;2*, *NRT1.1B*, *DEP1* and *PR1b*. TCP19 also interacts with PIL15 to co‐regulate the downstream genes. In addition, IDD10 interacted with both TCP19 and PIL15, inhibiting the activation of *DEP1*. Interestingly, DEP1 interacted with IDD10 to counteract its inhibitory effect, forming a negative feedback loop that fine‐tunes regulatory mechanism that modulates rice resistance to ShB. Notably, only TCP19 directly bound to and repressed *PR1b*, and its accumulation increased under HN conditions, further repressing *PR1b*. These findings uncover a regulatory network in which TCP19 and its interacting proteins coordinate nitrogen signalling and defence responses in rice.

## Results

### Identification of co‐regulated genes between N and *R. solani* in regulating transcriptomes

**Figure 1:**
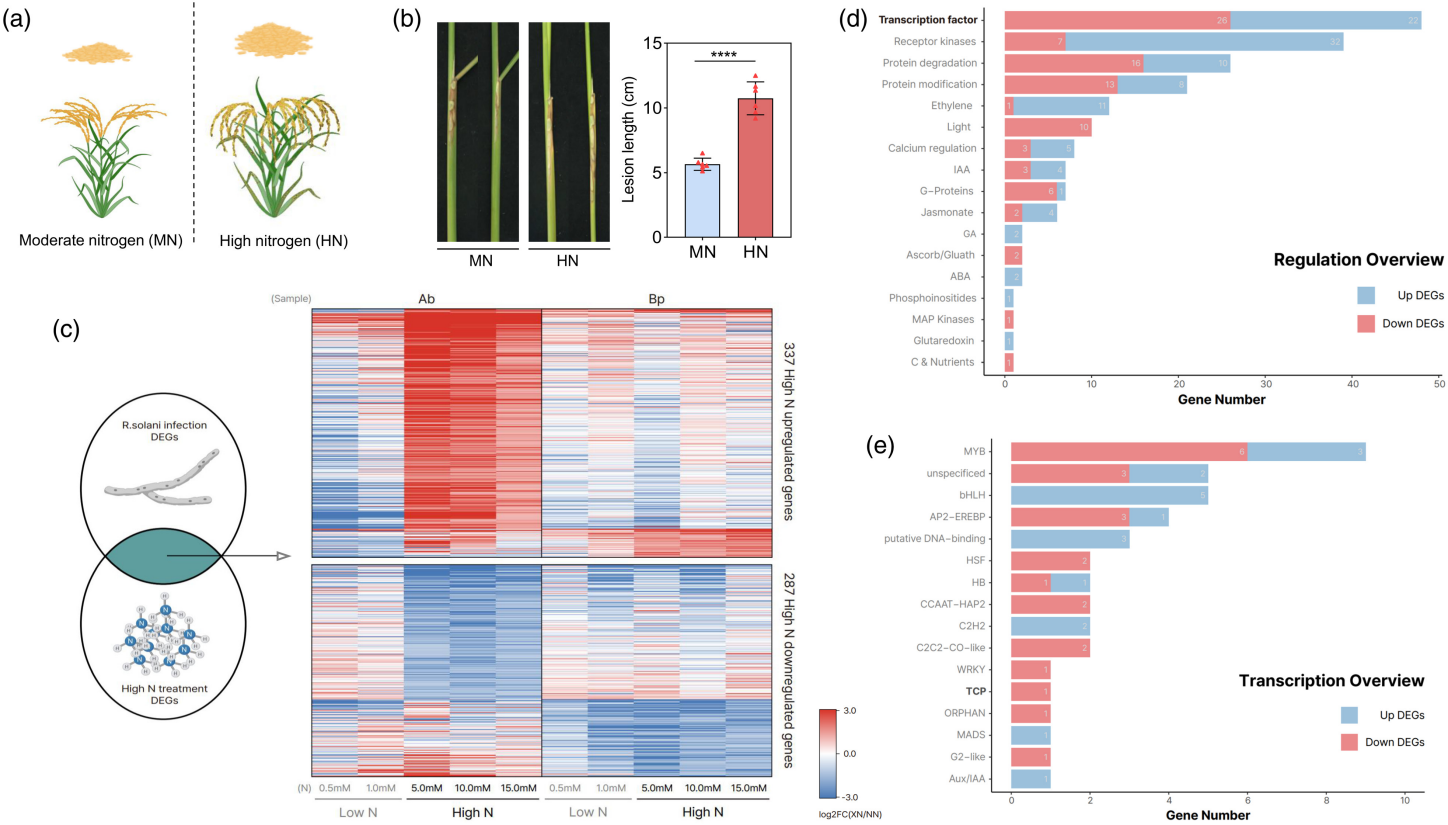
The incidence of ShB is associated with nitrogen fertilizer application. (a) Rice cultivated under high nitrogen application conditions yields more compared to rice grown under moderate nitrogen conditions. (b) Phenotypic observations and statistical analysis of disease incidence after *R. solani* inoculation of rice grown under high nitrogen and moderate nitrogen application conditions. Significant differences between groups were analysed using *t*‐tests. Asterisks indicate significant differences (*p* < 0.05). At least six replicates were used for each assay. Scale bar = 2 cm. (c) Heatmap analysis of common regulatory DEGs under *R. solani* infection and high N treatment. The colour gradient (red to blue) represents the strength of the log_2_ fold change values. Ab, axillary buds; Bp, basal parts of seedlings; NN, normal N treatment (2.0 mM); XN, five different concentrations of N treatment. (d, e) MapMan analysis of 337 upregulated DEGs and 287 downregulated DEGs, visualized in (d) regulation overview and (e) transcription overview.

The Green Revolution significantly increased agricultural productivity, accompanied by a substantial increase in nitrogen fertilizer application. Although excess N fertilizer can improve rice yield, it also leads to lower NUE and reduced immunity (Peng *et al*., 2010). Although higher N application enhanced rice yield (Figure 1a), it also increased the severity of the ShB incidence. ZH11 plants were cultivated with two urea concentrations: moderate nitrogen (MN, 150 kg/ha) and high nitrogen (HN, 300 kg/ha), followed by inoculation with *R. solani*. The results indicated that rice grown under HN conditions was more susceptible to ShB than that grown under MN conditions (Figure 1b). To explore the molecular mechanisms by which N signalling modulates ShB resistance, we examined the differentially expression of genes (DEGs) in response to the high‐nitrogen conditions associated with *R. solani* infection. A total of 624 DEGs were identified, with 337 upregulated and 287 downregulated genes (Figure 1c; Table S1) (Peng Yuan *et al*., 2020; Wang *et al*., 2020, 2021; Yang *et al*., 2015, 2022). The functional classification using MapMan analysis revealed that 31% (194/624) of the DEGs were involved in regulation, primarily associated with the bincode ‘transcription factor’ (Figure 1d), including MYB, bHLH, AP2/EREBP and TCP (Figure 1e).

### TCP19 negatively regulates ShB resistance

**Figure 2:**
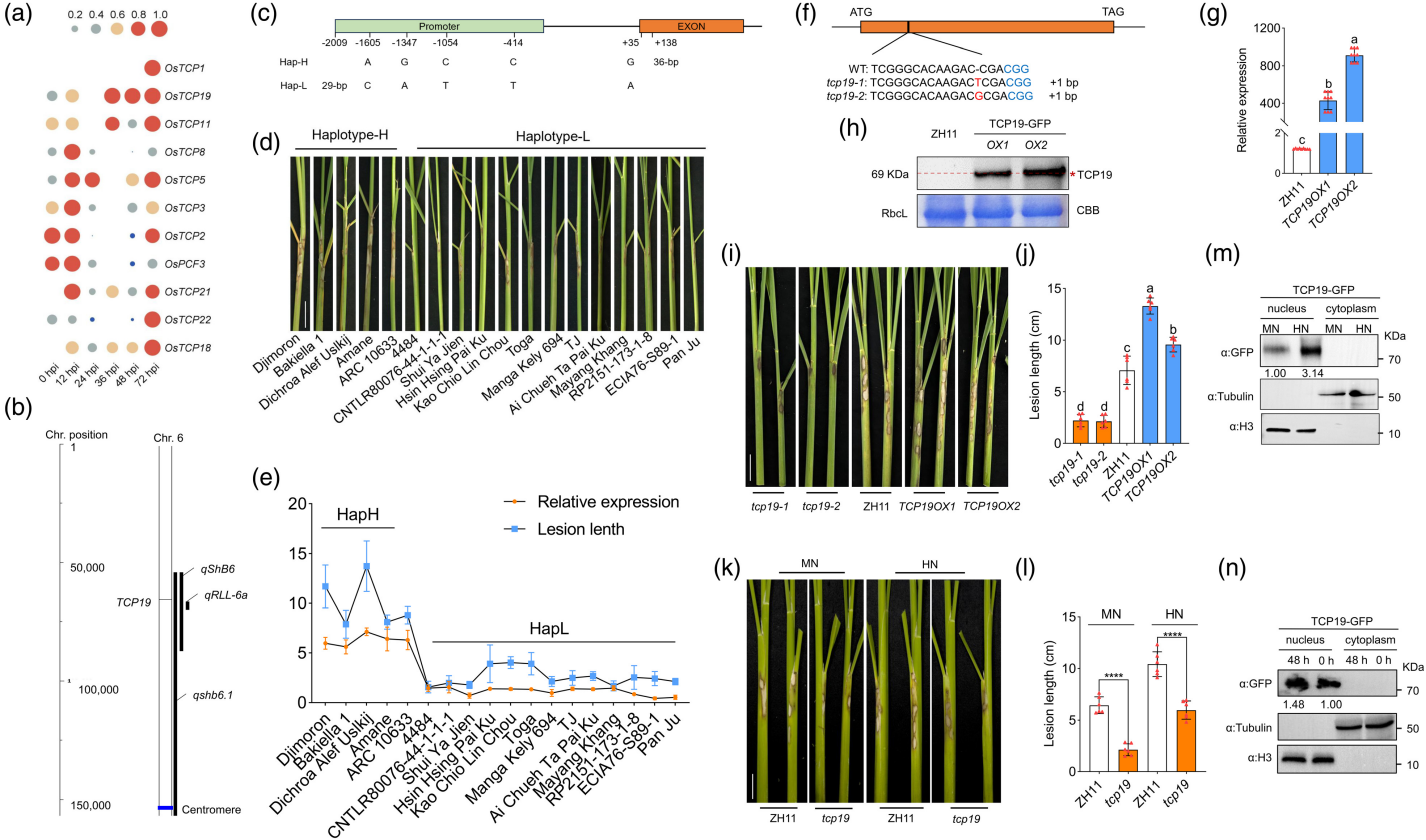
Identification of *TCP19* induction and its role in disease resistance induced by *R*. *solani*. (a) Heatmap of *TCP* family gene expression induced by *R. solani*. (b) *TCP19* co‐located with three quantitative trait loci (QTLs): *qShB6*, *qRLL‐6a* and *qshb6.1*. (c) SNPs and indels in Hap‐H and Hap‐L along with their genomic locations. (d) Phenotypic observations of incidence after *R. solani* inoculation in Hap‐H and Hap‐L. Scale bar = 2 cm. (e) Statistical analysis of disease spot length and relative expression of TCP19 in Hap‐H and Hap‐L. At least three replicates were used for each assay. (f) High‐throughput sequencing identification of *tcp19* CRISPR/Cas9 gene‐edited mutants, showing a 1‐bp insertion at the exon, leading to a frameshift. (g, h) WB and RT‐qPCR results of *TCP19* expression in overexpressed lines. Red asterisks indicate specific protein bands. At least three replicates were used for each RT‐qPCR assay. (i, j) Phenotypic observations and statistical analysis of incidence after *R. solani* inoculation in mutants, overexpression lines and WT. One‐way ANOVA was used to analyse significant differences between groups. Different lowercase letters above the bars indicate significant differences (*p* < 0.05). At least six replicates were used for each assay. Scale bar = 2 cm. (k, l) Phenotypic observations and statistical analysis of incidence after *R. solani* inoculation in *tcp19* mutants and WT under MN and HN conditions. A *t*‐test was used to analyse significant differences between groups. Asterisks indicate significant differences (*p* < 0.05). At least six replicates were used for each assay. Scale bar = 2 cm. (m, n) WB analysis of nucleoplasmic separation from *TCP19 OX‐GFP* after MN and HN treatments and 48 h after *R. solani* infection. H3 served as a nuclear marker, and tubulin was used as a cytoplasmic marker to validate the fraction purity. GFP detection was used to assess TCP19 accumulation in different subcellular compartments.

The TCP family protein in rice comprises 24 annotated members (Danisman, 2016; Martin‐Trillo and Cubas, 2010) (Figure S1a). Transcriptome data revealed that numerous TFs were responsive to *R. solani* infection, with 10 TCP family genes (*TCP21*, *TCP18*, *TCP19*, *TCP11*, *TCP1*, *TCP22*, *TCP5*, *TCP8*, *TCP2*, *TCP3* and *PCF3*) induced by inoculation with *R*. *solani* (Peng Yuan *et al*., 2020; Yang *et al*., 2022), among which *TCP19* exhibited the highest induction (Figure 2a). Additionally, TCP family genes can respond sensitively to N supply in rice (Wang *et al*., 2020). The heatmap data validated the transcriptome data by examining the gene expression patterns following *R. solani* inoculation and N treatment, indicating that *TCP19* was induced by both *R. solani* infection and HN treatment (Figure 2a; Figure S1b). Notably, *TCP19*, a well‐known regulator of NUE signalling (Liu *et al*., 2021b), was located within the ShB QTLs *qShB6* (from a cross between *O. sativa* and *O. nivara* lines) (Eizenga *et al*., 2013) and *qshb6.1* (from a cross between BPT‐5204 and ARC10531) (Yadav *et al*., 2015). Additionally, it was situated near another ShB QTL, *qRLL‐6a* (from a cross between HH1B and RSB02) (Liu *et al*., 2014) (Figure 2b). The *TCP19* locus has two haplotypes, Hap‐H and Hap‐L, which are associated with the rice NUE response and exhibit a 35 bp addition and 29 bp deletion in the promoter region, respectively (Liu *et al*., 2021b) (Figure 2c). To assess the haplotype contributions to ShB resistance, five cultivars of Hap‐H and 13 cultivars of Hap‐L were inoculated with *R. solani* AG1‐IA. The results indicated that the Hap‐H cultivars were more susceptible than the Hap‐L cultivars (Figure 2d,e). Moreover, RT‐qPCR results demonstrated that *TCP19* expression levels were positively correlated with lesion length in both Hap‐H and Hap‐L cultivars (Figure 1e).

*TCP19* mutants were generated using the CRISPR/Cas9 system, and we identified two independent mutants, *tcp19‐1* and *tcp19‐2*, each with a 1‐bp insertion in the first exon (Figure 2f). Furthermore, *TCP19‐GFP* overexpression (*TCP19 OXs*) was generated in the ZH11 background. WB and RT‐qPCR analyses confirmed that *TCP19* expression was significantly higher in *TCP19 OXs* than in ZH11 (Figure 2g,h). Upon inoculation with *R. solani* AG1‐IA, the *tcp19* mutants exhibited reduced susceptibility, whereas *TCP19 OXs* were more susceptible to ShB than to ZH11 (Figure 2i,j), indicating that TCP19 negatively regulates rice resistance to ShB. To assess the role of TCP19 in N‐mediated ShB susceptibility, plants were grown under MN and HN conditions and inoculated with *R. solani* AG1‐IA. The results indicated that ShB severity was higher under HN conditions in both ZH11 and *tcp19*. However, *tcp19* was less susceptible than ZH11 under both HN and MN conditions (Figure 2k,l), suggesting that *TCP19* modulates the N‐mediated ShB susceptibility. To investigate whether N treatment and *R. solani* infection influenced the accumulation and localization of TCP19, nucleoplasmic separation was performed in *TCP19‐GFP* plants. WB results showed that neither MN nor HN treatment nor *R. solani* infection affected the nuclear localization of TCP19 (Figure 2m,n). However, both *R. solani* infection and HN treatment led to a higher accumulation of TCP19 compared with the mock and MN conditions, respectively (Figure 2m,n).

### TCP19 directly regulates *NRT1.1B*, *AMT1;2* and *DEP1* to modulate ShB resistance

TCP19 regulates NUE and tillering in rice by directly repressing *DLT1* (Liu *et al*., 2021b). RT‐qPCR analysis showed that *DLT* expression was higher in *tcp19* mutants and lower in *TCP19 OXs* than that in ZH11 (Figure S2a). To investigate the role of *DLT* in ShB resistance, CRISPR/Cas9‐mediated genome editing was used to generate *DLT* gene mutants. Sequencing revealed that the *dlt‐11* and *dlt‐23* mutants had 25 and 1 bp deletions in the first exon, respectively (Figure S2b). Following inoculation with *R. solani*, the *dlt* mutants exhibited reduced susceptibility to ShB compared to ZH11 (Figure S2c,d), suggesting that TCP19 may regulate ShB resistance through other signalling pathways.

**Figure 3:**
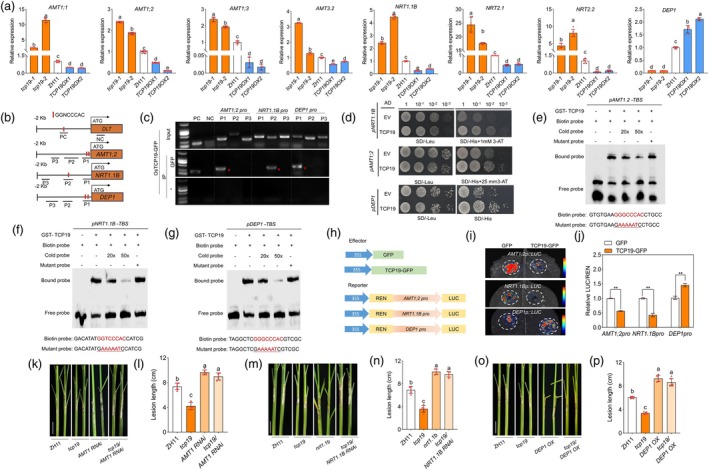
Screening and validation of TCP19 downstream genes. (a) Expression of nitrogen‐related genes (*AMT1;1*, *AMT1;2*, *AMT1;3*, *AMT3.2*, *NRT1.1B*, *NRT2.1*, *NRT2.2* and *DEP1*) downstream of TCP19 in *tcp19* mutants, overexpression lines and WT. One‐way ANOVA was used to analyse significant differences between groups. Different lowercase letters above the bars indicate significant differences (*p* < 0.05). At least six replicates were used for each assay. (b, c) ChIP–PCR analyses of *AMT1;2*, *NRT1.1B* and *DEP1* promoter regions. Red asterisks indicate specific DNA bands. (d) Y1H assay of TCP19 with *DEP1*, *NRT1.1B* and *AMT1;2* promoters. (e–g) EMSA to evaluate the binding interaction between TCP19 and the promoters of *AMT1;2*, *NRT1.1B* and *DEP1*. The labelled probe alone served as a negative control (Lane 1), and the labelled probe was incubated with TCP19‐GST showing a mobility shift due to protein–DNA binding (Lane 2). To verify the binding specificity, competitive binding assays were performed using two concentrations of cold probes (Lanes 3 and 4), which successfully competed with the labelled probe and reduced the intensity of the shifted band. A mutated version of the probe (Lane 5) showed no significant binding. (h–j) LUC reporter assays showed that TCP19 directly regulated the expression of *AMT1;2*, *NRT1.1B* and *DEP1*. Luciferase activities were shown relative to those obtained after transfection with 35S::GFP and the reporter construct, which was set to 1. A t‐test was used to analyse significant differences between groups. Asterisks indicate significant differences (*p* < 0.05). At least three replicates were used for each assay. (k, l) Phenotypic observations of incidence after *R. solani* inoculation and statistical analysis of disease spot length of *tcp19/AMT1 RNAi*, *tcp19*, *AMT RNAi* and WT. One‐way ANOVA was used to analyse significant differences between groups. Different lowercase letters above the bars indicate significant differences (*p* < 0.05). At least six replicates were used for each assay. Scale bar = 2 cm. (m, n) Phenotypic observations of incidence after *R. solani* inoculation and statistical analysis of disease spot length of *tcp19/NRT1.1B RNAi*, *tcp19*, *nrt1.1b* and WT. One‐way ANOVA was used to analyse significant differences between groups. Different lowercase letters above the bars indicate significant differences (*p* < 0.05). At least six replicates were used for each assay. Scale bar = 2 cm. (o, p) Phenotypic observations of incidence after *R. solani* inoculation and statistical analysis of disease spot length of *tcp19/DEP1 OX*, *tcp19*, *DEP1 OX* and WT. One‐way ANOVA was used to analyse significant differences between groups. Different lowercase letters above the bars indicate significant differences (*p* < 0.05). At least six replicates were used for each assay. Scale bar = 2 cm.

Because TCP19 regulates NUE, we investigated the expression levels of genes related to N uptake, metabolism and signalling in *tcp19* mutants and overexpressors. Specifically, we examined the expression of nitrate transporter proteins (*NRTs*), ammonium transporters (*AMTs*) and N assimilation pathway genes (*GS1;1*, *GS1;2*, *GDH1*, *GDH2* and *GOGAT1*). RT‐qPCR results indicated that the expression levels of *NRT1.1B*, *NRT2.1*, *NRT2.2*, *AMT1;1*, *AMT1;2*, *AMT1;3* and *AMT3.2* were higher in the *tcp19* mutants and lower in *TCP19 OXs* than in ZH11 (Figure 3a). Additionally, *DEP1*, a key NUE‐regulating gene, was expressed at lower levels in the *tcp19* mutants and higher levels in *TCP19 OXs* than in ZH11 (Figure 3a). The expression levels of *GS1;1*, *GDH1* and *GOGAT1* were consistent across *tcp19* mutants, overexpression lines and ZH11 (Figure S2e). However, in one *TCP19 OX* line and one *tcp19* mutant line, the expression levels of *GS1;2* and *GDH2* were significantly higher than those in ZH11, whereas in the other lines, these levels were comparable to those in ZH11. The expression of *NRT1.1A, GS1;2* and *GDH2* varied inconsistently between *tcp19* mutants and overexpression lines compared to ZH11 (Figure S2e). Previous studies have indicated that *nrt1.1b* and *AMT1 RNAi* lines are more susceptible to ShB than their wild‐type counterparts (Li *et al*., 2024a; Wu *et al*., 2022) (Figure S2f–i). In contrast, *dep1* mutants exhibited reduced susceptibility, while *DEP1 OXs* were more susceptible to ShB than to DJ (Liu *et al*., 2021a) (Figure S2j,k). These findings suggest that *TCP19* may regulate ShB resistance through modulation of *DEP1*, *NRT1.1B* and *AMT1*. To investigate whether TCP19 influenced nitrogen metabolites, the levels of two key amino acids, glutamate and glutamine, were measured. The results showed that the contents of glutamate and glutamine were similar in ZH11, *tcp19* and *TCP19 OXs* plants (Figure S3a,b). The nitrate content in *tcp19* mutants and *TCP19 OXs* was similar to that in ZH11 (Figure S3c). Additionally, methyl‐ammonium (MeA), a toxic ammonium analog, was tested to assess ammonium uptake. MeA treatment revealed that *tcp19* was more sensitive, whereas *TCP19 OX* was less sensitive to MeA than ZH11 (Figure S3d,e), suggesting that TCP19 inhibits ammonium uptake. N treatment transcriptome data was analysed (Wang *et al*., 2020). The results showed that the expression levels of *AMT1;1*, *AMT1;2* and *AMT3;2* were downregulated under HN conditions, whereas *NRT1.1B* and *DEP1* expression levels were upregulated (Figure S3f). These findings suggest that *AMTs*, *NRT1.1B* and *DEP1* may be involved in the TCP19‐regulated pathway.

Given that TCP19 is a transcription factor (TF), we investigated its direct regulation of *NRT1.1B*, *AMTs* and *DEP1* promoters. The predicted TCP19 binding motif, GGNCCCAC, was identified using the JASPAR 2024 database (https://jaspar.genereg.net/) (Rauluseviciute *et al*., 2024) (Figure S3g). Analysis of the promoter sequences revealed that putative TCP19‐binding motifs were present in the promoters of *NRT1.1B*, *AMT1s* and *DEP1* (Figure S3h). ChIP‐PCR results showed a significant enrichment of amplicons from P1 of the *AMT1;2* and *DEP1* promoters and P2 of the *NRT1.1B* promoter, which contained the GGNCCCAC motif, compared to the control (Figure 3b,c). Yeast one‐hybrid (Y1H) assay results demonstrated that TCP19 directly activated the promoters of *NRT1.1B*, *AMT1;2* and *DEP1* (Figure 3d), but not those of *AMT1;1* and *AMT1;3* (Figure S3i,j). To further confirm the direct binding of TCP19 to the promoters of *AMT1;2*, *NRT1.1B* and *DEP1*, an electrophoretic mobility shift assay (EMSA) was performed. The results showed that the GST‐TCP19 recombinant protein directly bound to the GGNCCCAC motifs in the promoters of *AMT1;2*, *NRT1.1B* and *DEP1* (Figure 3e–g). Transient assays with co‐expression of *TCP19* and *pDEP1‐LUC*, *pNRT1.1B‐LUC* or *pAMT1;2‐LUC* indicated that TCP19 directly activated *DEP1* but repressed *NRT1.1B* and *AMT1;2* (Figure 3h–j). To determine whether *AMT1;2*, *NRT1.1B* and *DEP1* function downstream of TCP19, we silenced or overexpressed these genes individually in a *tcp19* mutant background. The inoculation with *R. solani* demonstrated that *tcp19/AMT1;2 RNAi, tcp19/NRT1.1B RNAi* and *tcp19/DEP1 OX* lines presented increased susceptibility compared to ZH11 and *tcp19* (Figure 3k–p). These results confirmed that *AMT1;2*, *NRT1.1B* and *DEP1* were involved in the TCP19‐mediated regulation of ShB resistance.

### PIL15 interacts with TCP19 to negatively regulates ShB resistance

**Figure 4:**
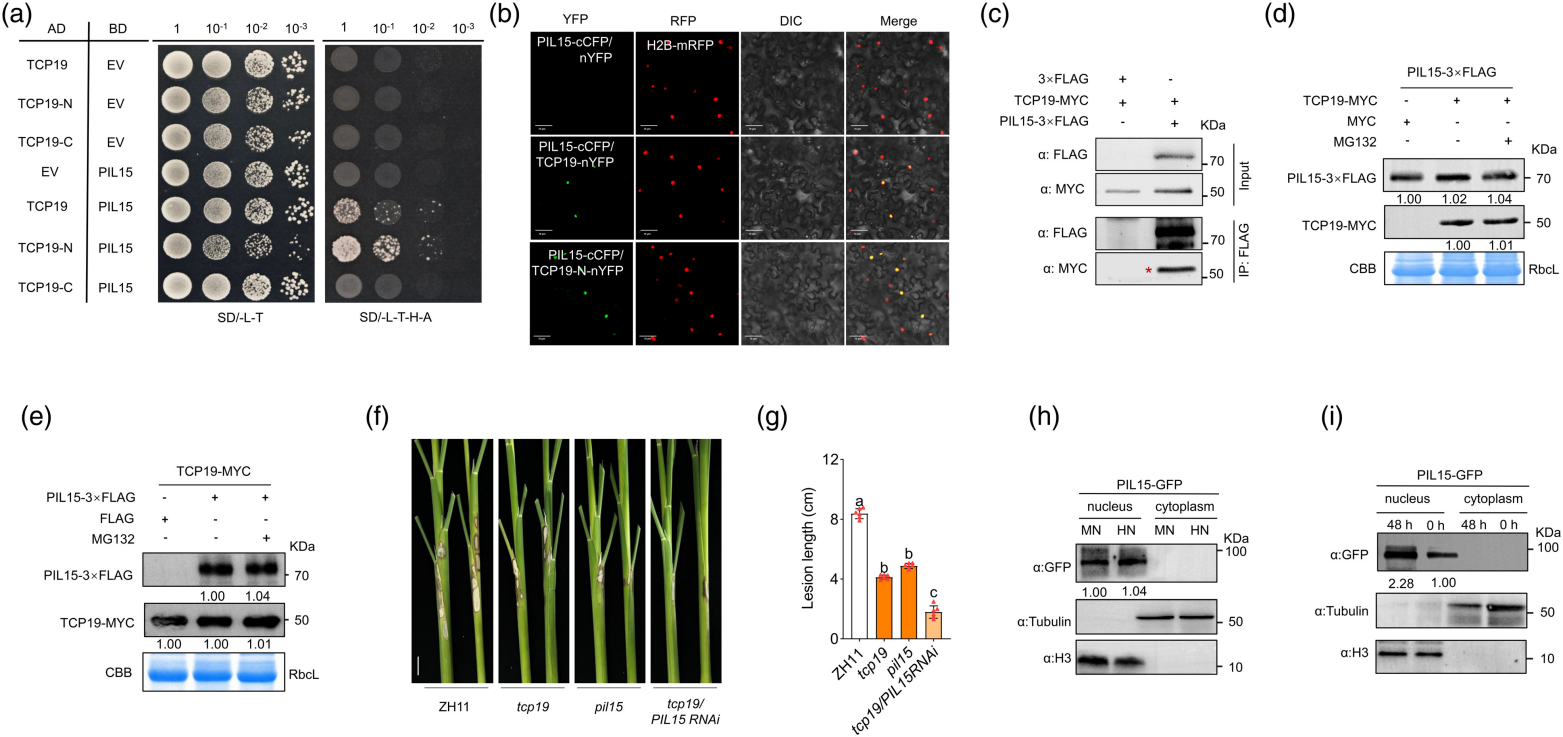
TCP19 interacted with PIL15. (a) Y2H assay showed that the N‐terminal of TCP19 interacted with PIL15 in yeast cells. Diploid yeast cells containing both TCP19‐AD and PIL15‐BD were grown in selective medium, whereas those in the negative control groups did not grow. EV, empty vector. (b) BiFC assay showed that TCP19 interacted with PIL15 in the nucleus. Co‐expression of TCP19‐nYFP or TCP19‐N‐nYFP, PIL15‐cCFP and H2B‐mRFP in tobacco leaves reconstructed functional YFP and co‐located with H2B‐mRFP, whereas the negative control groups did not show yellow fluorescence. Scale bar = 50 μm. (c) Co‐IP assay showed TCP19 interaction with PIL15. Co‐expression of TCP19‐MYC and PIL15‐3xFLAG in tobacco leaves was followed by immunoprecipitation using anti‐FLAG beads and subsequent WB using anti‐MYC and anti‐FLAG antibodies. Red asterisk indicates a specific protein band. IP, immunoprecipitation. (d, e) WB analysis of the impact of co‐expression on protein stability, with or without MG132 treatment (50 μM) to inhibit proteasomal degradation. (f, g) Phenotypic observations and statistical analysis of disease incidence after *R*. *solani* inoculation in *pil15* and *tcp19* mutants, *tcp19/PIL15RNAi* and WT. Significant differences between groups were analysed using one‐way ANOVA. Different lowercase letters above the bars indicate significant differences (*p* < 0.05). At least six replicates were used for each assay. Scale bar = 2 cm. (h, i) WB analysis of nucleoplasmic fractionation in *PIL15 OX‐GFP* under N treatment and 48 h after *R*. *solani* infection. H3 served as a nuclear marker, and tubulin was used as a cytoplasmic marker to validate the fraction purity. GFP detection was used to assess TCP19 accumulation in different subcellular compartments.

To explore the TCP19‐dependent mechanism of ShB resistance, the TCP19‐interacting proteins were screened. Structural prediction of TCP19 revealed a typical bHLH domain within the N‐terminal region spanning amino acids 59–233 (Figure S4a). Accordingly, TCP19 was divided into two segments: TCP19‐N (1–233 amino acids) and TCP19‐C (233–387 amino acids) (Figure S4a). Protein interaction assays were then performed. Using the Y2H library screening approach, TCP19 interactors were isolated, including PIL15. Y2H assays demonstrated that only the TCP19 N‐terminus interacted with PIL15 (Figure 4a). BiFC results further confirmed that TCP19 interacted with PIL15 in the nucleus and colocalized with the nuclear marker H2B‐mRFP (Figure 4b). Co‐IP assays indicated that TCP19‐MYC interacted with PIL15‐FLAG in planta (Figure 4c). To determine whether the interaction between TCP19 and PIL15 affects protein stability, plants were treated with 50 μM MG132, and the accumulation of TCP19 and PIL15 was assessed. WB results showed that TCP19 and PIL15 did not influence each other's protein levels (Figure 4d,e). To assess the role of *PIL15* in ShB resistance, mutant and overexpression lines of *PIL15* were generated. Sequencing revealed that the CRISPR/Cas9‐mediated mutants *pil15‐13* and *pil15‐14* had a 3‐bp deletion and a 1‐bp addition in the first exon, respectively (Figure S4b). WB and RT‐qPCR results indicated that *PIL15* expression was significantly higher in *PIL15 OXs* than in ZH11 (Figure S4c,d). Inoculation with *R. solani* AG1‐IA indicated that *pil15* mutants were less susceptible, whereas *PIL15 OXs* were more susceptible to ShB than WT (Figure S4e,f). This result is consistent with our previous findings for another *pil15* mutant, *PIL15‐RD2* (Yuan *et al*., 2023). Additionally, silencing of *PIL15* in the *tcp19* mutant background revealed that *tcp19/PIL15 RNAi* lines were less susceptible to ShB than both ZH11 and *tcp19* (Figure 4h,i). Furthermore, nucleoplasmic separation and WB results demonstrated that PIL15 levels increased upon *R. solani* infection but remained stable in the nucleus under HN treatment (Figure 4j,k).

**Figure 5:**
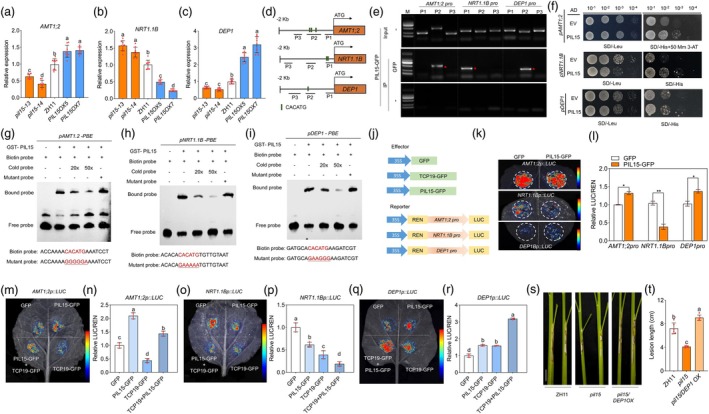
Analysis of TCP19 and PIL15 co‐regulation of downstream genes. (a–c) Expression levels of *NRT1.1B* and *AMT1;2* in *pil1*5 mutants, *PIL15OXs* and WT. (d, e) ChIP–PCR analyses of *AMT1;2*, *NRT1.1B* and *DEP1* promoter regions. Red asterisks indicate specific DNA bands. (f) Y1H assay showed that PIL15 interacted with *NRT1.1B*, *AMT1;*2 and *DEP1* promoters. (g–i) EMSA was used to evaluate the binding interaction between PIL15 and the promoters of *AMT1;2*, *NRT1.1B* and *DEP1*. (j–l) LUC reporter assays showed that TCP19 directly regulated the expression of *AMT1;2*, *NRT1.1B* and *DEP1*. Luciferase activities were shown relative to those obtained after transfection with 35S::GFP and the reporter construct, which was set to 1. A t‐test was used to analyse significant differences between groups. Asterisks indicate significant differences (*p* < 0.05). At least three replicates were used for each assay. (m–r) Co‐expression analysis demonstrating the regulatory effects of TCP19 and PIL15 on *AMT1;2*, *NRT1.1B* and *DEP1*. Luciferase activities were shown relative to those obtained after transfection with 35S::GFP and the reporter construct, which was set to 1. Significant differences between groups were analysed using one‐way ANOVA. Different letters above the bars indicate significant differences (*p* < 0.05). At least three replicates were used for each assay. (s, t) Phenotypic observations and statistical analysis of disease incidence after *R*. *solani* inoculation in *pil15* mutants, *pil15/DEP1 OX* and WT. Significant differences between groups were analysed using one‐way ANOVA. Different letters above the bars indicate significant differences (*p* < 0.05). At least six replicates were used for each assay. Scale bar = 2 cm.

The regulation of *PIL15* on *NRT1.1B*, *AMT1;2* and *DEP1* was investigated. RT‐qPCR results indicated that *NRT1.1B* expression levels were higher in the *pil15* mutants but lower in the *PIL15 OXs* than in WT, whereas *AMT1;2* and *DEP1* expression was lower in the *pil15* mutants and higher in *PIL15 OXs* than in WT (Figure 5a–c), indicating that *PIL15* activated *DEP1* and *AMT1;2* but repressed *NRT1.1B*. Promoter sequence analysis identified the putative PIL15‐binding motif PBE box (CACATG) (Li *et al*., 2022) in the promoter regions of *AMT1;2*, *NRT1.1B* and *DEP1* (Figure S4g). ChIP‐PCR results suggested that the amplicons of P2 from the *AMT1;2* and *DEP1* promoters and P1 from the *NRT1.1B* promoter, which contained the CACATG motif, were significantly enriched compared to controls (Figure 5d,e). Y1H assays demonstrated that PIL15 bound to the *AMT1;2*, *NRT1.1B* and *DEP1* promoters (Figure 5f). EMSA results demonstrated that PIL15 directly bound to the PBE motifs in the promoters of *AMT1;2*, *NRT1.1B* and *DEP1* (Figure 5g–i). Transient assays involving the co‐expression of PIL15 with *pDEP1‐LUC, pAMT1;2‐LUC* or *pNRT1.1B‐LUC* revealed that PIL15 directly activated *DEP1* and *AMT1;2* but repressed *NRT1.1B* promoters (Figure 5j–l). To further investigate the effects of the TCP19‐PIL15 complex on *AMT1;2*, *NRT1.1B* and *DEP1* expression, transient assays and RT‐qPCR were performed. The results demonstrated that TCP19 partially inhibited the PIL15‐mediated activation of *AMT1;2*. Moreover, the TCP19‐PIL15 complex enhanced the repression of *NRT1.1B* and increased the activation of *DEP1* (Figure 5m–r, Figure S4h–j). Additionally, *DEP1* was overexpressed in the *pil15* mutant background. Inoculation with *R. solani* demonstrated the increased susceptibility of *pil15/DEP1 OX* compared with ZH11 and *pil15* (Figure 5s,t). In addition, based on the ChIP‐PCR results (Figures 3b,c, 5d,e), TCP19 and PIL15 cannot enrich each other's binding regions on the target gene promoters. To further verify this mode of regulation under nitrogen treatment, we performed additional ChIP‐PCR assays by HN treatment. Similar results were observed, as TCP19 and PIL15 did not show enrichment at the same promoter regions (Figure S4k–m).

### IDD10 interacts and inhibits TCP19‐PIL15 dependent repression of *DEP1*

**Figure 6:**
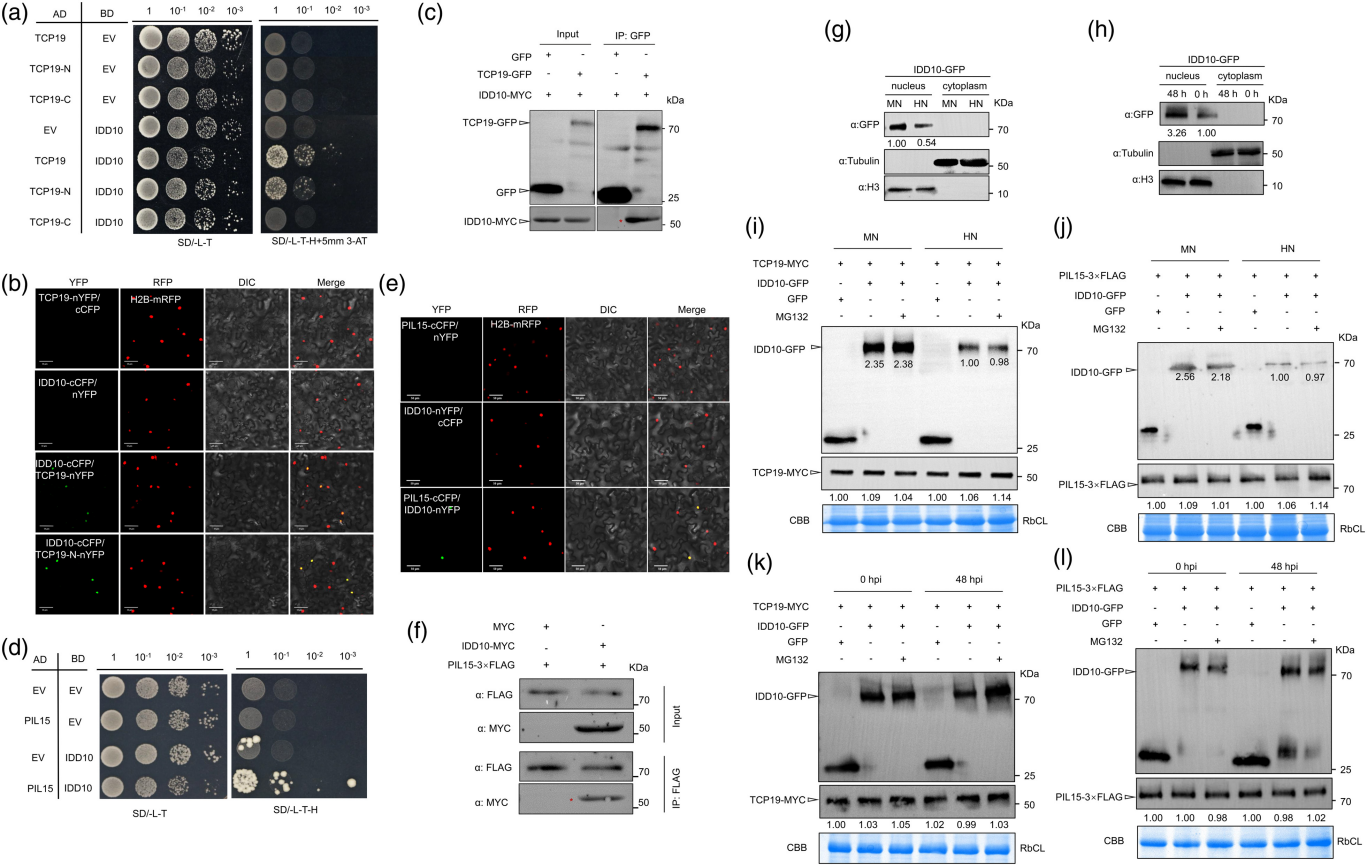
TCP19, PIL15 and IDD10 interacted with each other. (a) Y2H assay showed that the TCP19‐N terminal interacted with IDD10 in yeast cells. Diploid yeast cells containing both TCP19‐AD and IDD10‐BD were grown in selective medium, whereas the negative control groups did not grow. EV, empty vector. (b) BiFC assay showed that TCP19 interacted with IDD10 in the nucleus. Co‐expression of TCP19‐nYFP, IDD10‐cCFP and H2B‐mRFP in tobacco leaves reconstructed functional YFP and co‐located with H2B‐mRFP, whereas the negative control groups did not show yellow fluorescence. Scale bar = 50 μm. (c) CoIP assay showed an interaction between TCP19 and IDD10. Co‐expression of TCP19‐GFP and IDD10‐MYC in tobacco leaves was followed by immunoprecipitation with anti‐GFP beads and a subsequent WB assay with anti‐GFP and anti‐MYC antibodies. Red asterisk indicates a specific protein band. IP, immunoprecipitation. (d) Y2H assay showed that PIL15 interacted with IDD10 in yeast cells. Diploid yeast cells containing both PIL15‐AD and IDD10‐BD were grown in selective medium, whereas the negative control groups did not grow. EV, empty vector. (e) BiFC assay showed that PIL15 interacted with IDD10 in the nucleus. Co‐expression of IDD10‐nYFP, PIL15‐cCFP and H2B‐mRFP in tobacco leaves reconstructed functional YFP and co‐located with H2B‐mRFP, whereas the negative control groups did not show yellow fluorescence. Scale bar = 50 μm. (f) CoIP assay showed that PIL15 interacted with IDD10. Co‐expression of PIL15‐3xFLAG and IDD10‐MYC in tobacco leaves was followed by immunoprecipitation with anti‐FLAG beads and subsequent WB assay with anti‐FLAG and anti‐MYC antibodies. IP, immunoprecipitation. (g, h) WB analysis of nucleoplasmic fractionation in *IDD10 OX‐GFP* under N treatment and 48 h after *R. solani* infection. H3 served as a nuclear marker, and tubulin was used as a cytoplasmic marker to validate the fraction purity. GFP detection was used to assess IDD10 accumulation in different subcellular compartments. (i–l) WB analysis was performed to assess the effect of IDD10 on the stability of TCP19 and PIL15 under N treatment and 48 h after *R. solani* infection. Protein stability was evaluated with and without MG132 treatment (50 μM), which inhibited proteasomal degradation.

**Figure 7:**
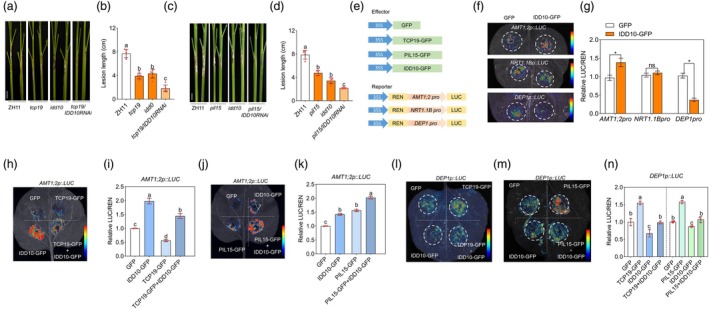
(a, b) Phenotypic observations of incidence after *R. solani* inoculation and statistical analysis of disease spot length of *tcp19*, *idd10* mutants, *tcp19/IDD10 RNAi* and WT. One‐way ANOVA was used to analyse significant differences between groups. Different lowercase letters above the bars indicate significant differences (*p* < 0.05). At least six replicates were used for each assay. Scale bar = 2 cm. (c, d) Phenotypic observations of incidence after *R. solani* inoculation and statistical analysis of disease spot length of *pil15*, *idd10* mutants, *pil15/IDD10 RNAi* and WT. One‐way ANOVA was used to analyse significant differences between groups. Different lowercase letters above the bars indicate significant differences (*p* < 0.05). At least six replicates were used for each assay. (e–g) LUC reporter assays showed that IDD10 regulated the expression of *AMT1;2* and *DEP1*, but not *NRT1.1B*. Luciferase activities were shown relative to those obtained after transfection with 35S::GFP and the reporter construct, which was set to 1. A t‐test was used to analyse significant differences between groups. Asterisks indicate significant differences (*p* < 0.05). At least three replicates were used for each assay. (h–k) Co‐expression analysis revealed the regulatory effects of IDD10 and TCP19/PIL15 on *AMT1;2*. Luciferase activities were shown relative to those obtained after transfection with 35S::GFP and the reporter construct, which was set to 1. Significant differences between groups were analysed using one‐way ANOVA. Different lowercase letters above the bars indicate significant differences (*P* < 0.05). At least three replicates were used for each assay. (l–n) Co‐expression of IDD10 and TCP19 or PIL15 inhibited TCP19‐ or PIL15‐mediated activation of *DEP1*. Luciferase activities were shown relative to those obtained after transfection with 35S::GFP and the reporter construct, which was set to 1. One‐way ANOVA or t‐test was used to analyse significant differences between groups. Different lowercase letters above the bars indicate significant differences (*p* < 0.05). At least three replicates were used for each assay.

In Y2H screening, IDD10 was identified as another protein interacting with TCP19. Y2H results indicated that IDD10 interacted with the N‐terminal, but not the C‐terminal, of TCP19 (Figure 6a). BiFC assays demonstrated that IDD10 interacted with the N‐terminus of TCP19 in the nucleus, with the co‐expression of TCP19‐nYFP or TCP19‐N‐nYFP, IDD10‐cCFP and H2B‐mRFP in tobacco leaves, resulting in functional YFP reconstruction, which co‐localized with H2B‐mRFP (Figure 6b). Co‐IP assays further confirmed the interaction between TCP19 and IDD10 in plants (Figure 6c). Additionally, since TCP19 interacted with PIL15, the interaction between IDD10 and PIL15 was examined, revealing through Y2H, BiFC and co‐IP assays that IDD10 interacted with PIL15 (Figure 6d–f). To determine whether the localization and accumulation of IDD10 were altered by N treatment and *R*. *solani* infection, nucleoplasmic separation and WB analysis were performed. These results indicated that the localization of IDD10 remained unchanged under these stimuli. However, IDD10 protein levels in the nucleus were increased by *R*. *solani* infection and reduced by HN treatment (Figure 6g,h). Subsequently, to elucidate the mechanism of interaction between IDD10, TCP19 and PIL15, we assessed the effect of IDD10 on the stability of PIL15 and TCP19 under N treatments and *R*. *solani* infection conditions. The results showed that IDD10 did not affect the stability of TCP19 or PIL15 under these conditions (Figure 6i–l). A previous study has shown that *IDD10* negatively regulates ShB (Li *et al*., 2024b). Silencing of *IDD10* in the *tcp19* and *pil15* mutant backgrounds revealed that *tcp19*/*IDD10 RNAi* and *pil15*/*IDD10 RNAi* lines were less susceptible to *R*. *solani* infection than ZH11, *tcp19* or *pil15* (Figure 7a–d).

IDD10 activates the expression of ammonium‐dependent genes and directly activates *AMT1;2* (Xuan *et al*., 2013). The expression levels of *DEP1* and *NRT1.1B* were similar among WT, *idd10* and *IDD10 OX* plants (Figure S5a,b). Furthermore, transient assays demonstrated that IDD10 did not regulate *NRT1.1B* expression but inhibited *DEP1* expression (Figure 7e–g). Next, transient assays were conducted to analyse IDD10's function in *TCP19* and *PIL15* transcription. The results showed that IDD10 did not directly regulate *TCP19* or *PIL15* transcription (Figure S5c–f). Promoter sequence analysis revealed the presence of an IDD10 binding motif in the *NRT1.1B* and *AMT1;2* promoters (Figure S5g), whereas IDD10 did not regulate *NRT1.1B* transcription. Therefore, *NRT1.1B* may be co‐regulated by TCP19 and PIL15 rather than by IDD10. The regulation of *DEP1* by IDD10 may involve a more complex mechanism, as putative IDD10‐binding motifs (TTTGTCC/G) were not present in the *DEP1* promoter (Figure S5g). Previous studies have shown that IDD family proteins, such as JKD, activate *SCR* and *MGP* promoters without the putative IDD‐binding motifs (Ogasawara *et al*., 2011), suggesting alternative binding motifs for IDDs. However, Y1H assays indicated that IDD10 did not interact with the *DEP1* promoter (Figure S5h). To investigate the mechanisms by which IDD10‐TCP19 and IDD10‐PIL15 regulate *AMT1;2* and *DEP1*, transient assay results revealed that IDD10 enhanced PIL15‐mediated activation of *AMT1;2*, while TCP19 partially attenuated IDD10‐induced activation of *AMT1;2* (Figure 7h–k). Additionally, co‐expression of IDD10 with TCP19 or PIL15 inhibited the TCP19‐ or PIL15‐mediated activation of *DEP1* (Figure 7l–n). These results indicated that IDD10 neither directly affect the stability of TCP19 or PIL15, nor did it act as a direct upstream regulator of these two genes. This suggests that their interactions likely occur through the regulation of downstream genes. IDD10 and TCP19 exhibited opposite regulatory effects on *AMT1;2*, while both IDD10 and PIL15 functioned as activators, demonstrating a synergistic relationship in regulating *AMT1;2*. Furthermore, IDD10 did not participate in the regulation of *NRT1.1B*. Additionally, its interaction with TCP19 and PIL15 partially suppressed their activation of *DEP1*.

### DEP1 interacts with IDD10 to release TCP19‐PIL15

**Figure 8:**
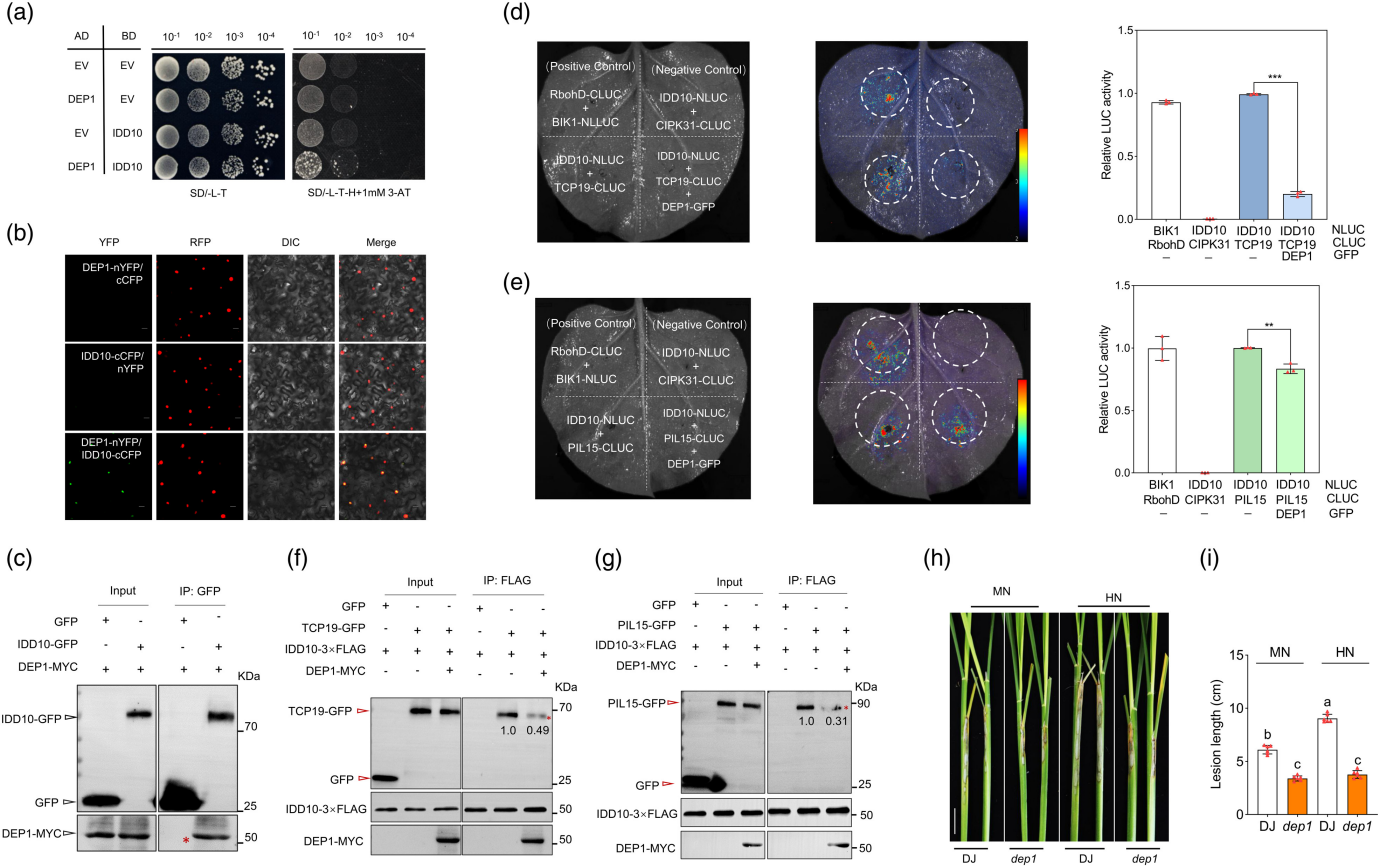
TCP19 and PIL15 activated *DEP1* and DEP1 interactions with IDD10 to release TCP19 and PIL15. (a) Y2H assay showed that DEP1 interacted with IDD10 in yeast cells. Diploid yeast cells containing both DEP1‐AD and IDD10‐BD were grown in selective medium, whereas the negative control groups did not grow. EV, empty vector. (b) BiFC assay showed that DEP1 interacted with IDD10 in the nucleus. Co‐expression of DEP1‐nYFP, IDD10‐cCFP and H2B‐mRFP in tobacco leaves reconstructed functional YFP and co‐located with H2B‐mRFP, whereas the negative control groups did not show yellow fluorescence. Scale bar = 50 μm. (c) CoIP assay showed that DEP1 interacted with IDD10. Co‐expression of DEP1‐MYC and IDD10‐GFP in tobacco leaves was followed by immunoprecipitation with anti‐GFP beads and a subsequent WB assay with anti‐GFP and anti‐MYC antibodies. Red asterisk indicates a specific protein band. IP, immunoprecipitation. (d, e) Co‐expression of DEP1 with IDD10 and TCP19 or PIL15 showed that DEP1 inhibited the interaction between IDD10 and TCP19 or PIL15. Luciferase activities were shown relative to those obtained after transfection with 35S::GFP and the reporter construct, which was set to 1. A t‐test was used to analyse significant differences between with or without DEP1‐GFP groups. Asterisks indicate significant differences (*p* < 0.05). At least three replicates were used for each assay. (f, g) Competitive co‐IP assays showed that DEP1 inhibited the interaction between IDD10 and TCP19 or PIL15. IDD10‐FLAG was co‐expressed with TCP19‐GFP or PIL15‐GFP in the presence or absence of DEP1‐MYC. Immunoprecipitation was performed using anti‐FLAG antibodies, followed by immunoblotting to detect IDD10, TCP19, DEP1 and PIL15. IP, immunoprecipitation. (h, i) Phenotypic observations and statistical analysis of incidence after *R. solani* inoculation in *dep1* mutants and WT under MN and HN conditions. One‐way ANOVA was used to analyse significant differences between groups. Different letters above the bars indicate significant differences (*p < 0.05*). At least three replicates were used for each assay. Scale bar = 2 cm.

Previously, it was shown that DEP1 could interact with LPA1/IDD14 to inhibit the activation of PIN1a, thus negatively regulating rice ShB resistance (Liu *et al*., 2021b) (Figure S2j,k). Because IDD10 and LPA1/IDD14 are members of the IDD family, the interaction between IDD10 and DEP1 was investigated. Y2H assays revealed that IDD10 interacted with DEP1 (Figure 8a), and BiFC assays confirmed this interaction in the nucleus (Figure 8b). Co‐IP assays further validated the interaction between IDD10 and DEP1 in tobacco (Figure 8c). However, Y2H results indicated that DEP1 did not interact with TCP19 or PIL15 (Figure S6a,b).

To investigate whether the interaction between DEP1 and IDD10 influenced the release of TCP19 and PIL15, a yeast three‐hybrid assay (Y3H) was conducted. These results demonstrated a strong interaction between IDD10 and TCP19 or PIL15 in the absence of DEP1 expression. However, DEP1 expression significantly reduced the interaction strength between IDD10 and TCP19 or PIL15 (Figure S6c–e). Additionally, the Split Firefly Luciferase Complementation (SFLC) assay results confirmed that DEP1 inhibited the interaction between IDD10 and TCP19 or PIL15 (Figure 8d,e). Competitive co‐IP assays were then performed, in which DEP1‐MYC was added to a reaction containing IDD10‐FLAG and TCP19‐GFP or PIL15‐GFP. The results showed that the presence of DEP1 significantly reduced the interaction affinity between IDD10 and TCP19 or PIL15 (Figure 8f,g). These findings clearly indicated that DEP1 inhibited the interaction between IDD10 and TCP19 or PIL15, suggesting that DEP1 suppresses IDD10 through a feedback mechanism. Finally, to determine the pathway by which TCP19 confers resistance to ShB under HN conditions, we revisited our previous findings. The two downstream targets of TCP19, *AMT1* and *NRT1.1B*, showed contrasting roles in ShB resistance under HN conditions. Specifically, *AMT1 OX* exhibited ShB resistance under MN conditions but lost resistance under HN conditions, whereas *nrt1.1b* exhibited enhanced susceptibility under HN conditions (Li *et al*., 2023; Wu *et al*., 2022). Additionally, the *dep1* mutant's response to ShB under MN and HN conditions was examined. The results showed that *dep1* was less susceptible to ShB under both MN and HN conditions than the wild type, and *dep1* susceptibility was not enhanced under HN conditions (Figure 8h,i). These results suggest that TCP19 likely promotes ShB resistance under HN conditions by activating *DEP1* and repressing *NRT1.1B*.

### TCP19 regulates high nitrogen suppression of *PR1b* expression

**Figure 9:**
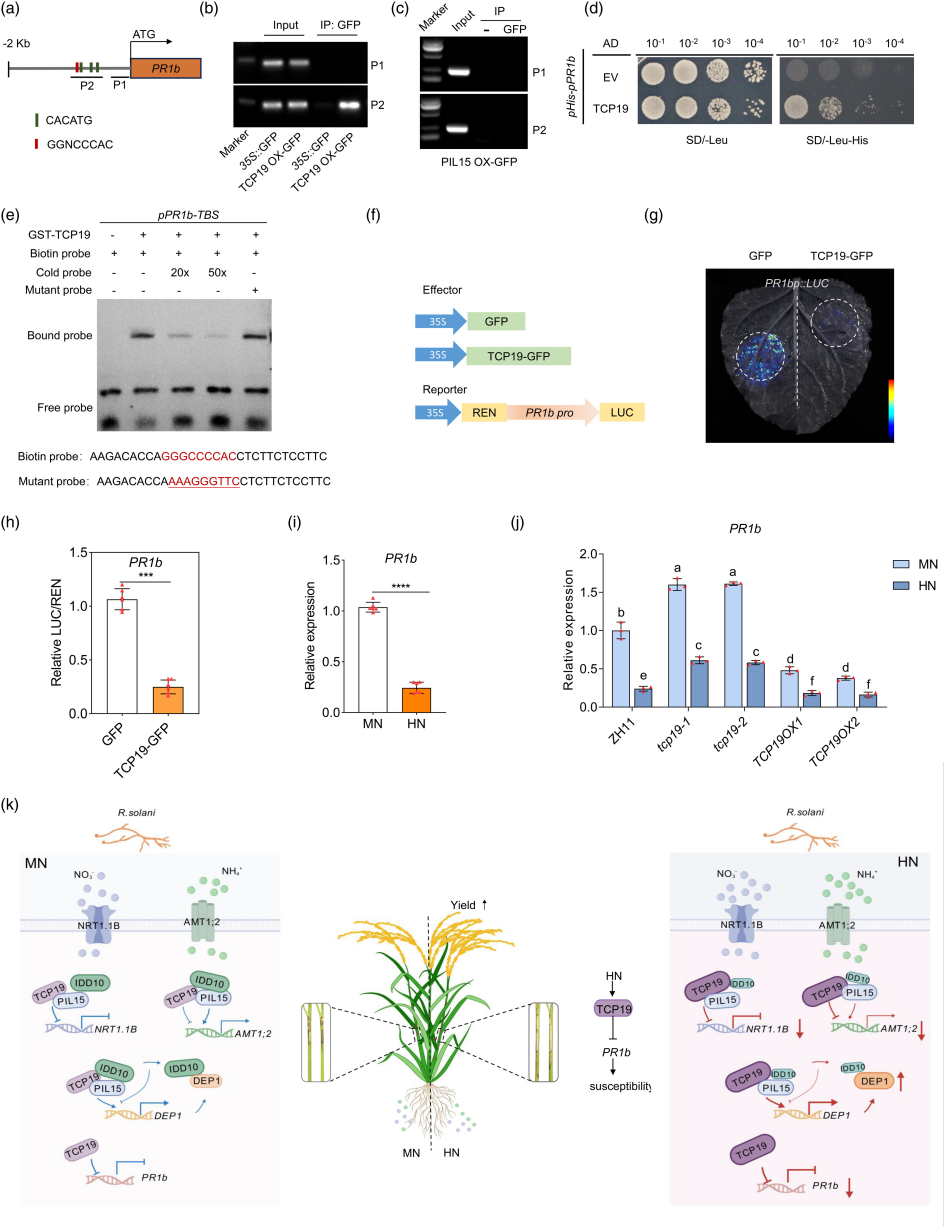
TCP19 directly represses *PR1b* expression. (a–c) ChIP–PCR analyses of *PR1b* promoter regions in *TCP19‐GFP OX* and *PIL15‐GFP OX* transgenic rice. (d) Y1H assay of TCP19 with the promoter of *PR1b*. (e) EMSA to evaluate the binding interaction between TCP19 and the promoters of *PR1b*. (f) LUC reporter assays showed that TCP19 directly regulated the expression of *PR1b*. Luciferase activities were shown relative to those obtained after transfection with *35S::GFP* and the reporter construct, which was set to 1. A t‐test was used to analyse significant differences between groups. Asterisks indicate significant differences (*p < 0.05*). At least three replicates were used for each assay. (i) Expression levels of *PR1b* under MN and HN conditions. (j) Expression levels of *PR1b* in *tcp19* mutants, overexpressions and WT under MN and HN conditions. (k) Proposed working model of TCP19‐mediated regulatory mechanisms in rice and under *R. solani* infection and different N conditions (MN vs HN). Under both MN and HN conditions, *R. solani* infection induces the expression of TCP19, PIL15 and IDD10, but to different extents depending on the N level. TCP19 interacts with IDD10 and PIL15 to regulate the expression of nitrogen uptake and nitrogen signalling genes, including *AMT1;2*, *NRT1.1B* and *DEP1*. Specifically, TCP19 suppresses *AMT1;2* expression, whereas the PIL15‐IDD10 complex synergistically activates *AMT1;2*. Differently, TCP19 and PIL15 together repress *NRT1.1B* expression, while IDD10 does not regulate *NRT1.1B*. TCP19 and PIL15 synergistically activate *DEP1*, whereas IDD10 interacts with TCP19 and PIL15 to attenuate the activation of *DEP1*. Additionally, DEP1 exerts feedback regulation by interacting with IDD10, thereby weakening IDD10's interaction with TCP19 and PIL15 to further balance nitrogen signalling. Under HN conditions, *TCP19* expression is upregulated, whereas IDD10 expression is downregulated, leading to stronger activation of *DEP1*. In addition, TCP19 directly suppresses the expression of *PR1b*, thereby negatively regulating resistance to ShB. PIL15 and IDD10 are not involved in this regulatory pathway. Notably, the repressive effect of TCP19 on *PR1b* is further enhanced under HN conditions, which contributes to increased susceptibility to ShB. The size and colour intensity of the protein icons represent their expression levels, with larger and darker icons indicating higher expression. Both arrows thickness and colours represent the relative intensity of the activation or inhibition between components, with thicker lines indicating stronger regulatory effects. Blue arrows denote regulatory relationships under MN, while red arrows indicate those under HN conditions. Upward (↑) and downward (↓) arrows are used to indicate activation or repression of downstream target genes, respectively.

To elucidate the direct cause of increased susceptibility to ShB under HN conditions, TCP19 regulation on *pathogenesis‐related* (*PR*) genes was investigated. Promoter sequence analysis revealed the presence of putative TCP19 binding motifs in the *PR1b* promoter, but not in the promoters of *PR1a* or *PBZ1* (Figure S7a). To test the direct regulation of TCP19 to *PR1b*, ChIP‐PCR was performed. The results confirmed that TCP19 specifically bound to the P2 region of the *PR1b* promoter, but PIL15 did not bind to the *PR1b* promoter (Figure 9a–c). Y1H assays further demonstrated that TCP19 directly interacts with the *PR1b* promoter, while no interaction was observed with the *PR1a* or *PBZ1* promoters (Figure 9d; Figure S7b). EMSA analysis revealed that TCP19 specifically binds to a probe containing the GGGCCCAC motif (Figure 9e). Furthermore, LUC reporter assays showed that TCP19 represses *PR1b* expression (Figure 9f–h). These results demonstrate that TCP19 directly binds to the *PR1b* promoter and represses its transcription. To further determine whether *PR* genes directly contribute to the reduced ShB resistance under HN conditions, the expression levels of *PR1b*, *PR1a* and *PBZ1* were examined under MN and HN conditions. RT‐qPCR analysis revealed that *PR1b* expression was significantly downregulated under HN conditions, while *PR1a* was induced, and *PBZ1* expression remained unchanged (Figure 9i; Figure S7c,d). We further analysed *PR* gene expression in *tcp19* mutants and *TCP19 OXs* under MN and HN conditions. The results showed that TCP19‐mediated repression of *PR1b* was enhanced under HN conditions (Figure 9j). Together, these findings suggest that HN suppresses *PR1b* expression through TCP19.

## Discussion

### Effects of nitrogen on plant growth and disease susceptibility

Since the Green Revolution, the widespread use of nitrogen fertilizers has significantly increased global agricultural production (Wu *et al*., 2020). As a crucial nutrient, nitrogen plays a crucial role in plant growth and development, influencing various aspects such as plant height, root development and overall yield. High nitrogen application not only promotes rapid growth and enhances chlorophyll content, thus improving photosynthetic efficiency, but also increases tillering capacity and overall plant productivity (Chen *et al*., 2022). However, along with the benefits of nitrogen fertilization, several negative effects have emerged, particularly with regard to plant disease susceptibility. Numerous studies have shown that excessive nitrogen application can exacerbate the incidence and severity of various plant diseases, including ShB in rice. Under high nitrogen conditions, disease spread often accelerates, with more severe symptoms leading to significant crop yield losses (Savary *et al*., 1995). Despite widespread observation of this phenomenon, the underlying mechanisms by which high nitrogen levels exacerbate disease susceptibility remain unclear. Understanding how nitrogen influences plant immunity and defence mechanisms to reduce disease resistance is a key area of ongoing research. Therefore, investigating the reasons behind the reduced disease resistance under high nitrogen conditions is critical, as it could enhance our understanding of plant–pathogen interactions and inform strategies for disease management in nitrogen‐rich agricultural systems.

### Identification of key genes and mechanisms through transcriptome analysis

To explore the molecular mechanisms underlying the effect of nitrogen on ShB resistance, we conducted comparative transcriptome analyses using *R*. *solani* inoculation and HN treatments. By integrating the DEGs identified in both transcriptomes, we identified a subset of genes consistently regulated under both conditions, suggesting their involvement in the N‐ShB regulatory network. Further MapMan analysis revealed that these DEGs were enriched in several pathways, including receptor kinases, protein degradation, ethylene, jasmonate and light signalling, which are key processes known to influence plant growth and defence responses (Ballaré, 2014; Bari and Jones, 2009; Santino *et al*., 2013). Notably, transcription factor families such as MYB, bHLH, AP2/EREBP and WRKY were prominently represented, underscoring their potential roles in coordinating defence signalling pathways (Erpen *et al*., 2018). Among them, the TCP family emerged as a particularly interesting candidate. Although less represented than other families, its significant regulation under both nitrogen and *R*. *solani* conditions suggests a specialized function in modulating the crosstalk between N signalling and ShB resistance.

### Mechanisms by which high nitrogen exacerbates disease susceptibility

Within the TCP family, *TCP19* was strongly induced by both *R*. *solani* infection and N treatment. Recent studies have reported that TCP19 modulates N‐dependent tillering and NUE in rice (Liu *et al*., 2021b). Further analysis revealed that TCP19 was co‐located with three known ShB‐related QTLs (*qShB6*, *qShB6.1* and *qRLL‐6a*), and haplotype analysis combined with mutant inoculation experiments confirmed that *TCP19* negatively regulated ShB resistance. These findings suggest that TCP19 may be a key regulator of N‐ShB signalling. Subsequent analyses identified several N uptake and signalling genes, including *AMT1;2*, *NRT1.1B* and *DEP1*, as direct targets of TCP19, which is involved in the regulation of ShB resistance. This was confirmed through genetic combinations of *TCP19* with *AMT1;2*, *NRT1.1B* and *DEP1*. *DEP1*, a crucial regulator of tillering and panicle structure in rice (Huang *et al*., 2009, 2022; Zhao *et al*., 2019), negatively affected ShB resistance (Liu *et al*., 2021b) (Figure S2j,k). In contrast, *NRT1.1B* and *AMT1;2*, which are involved in nitrogen uptake in rice (Hu *et al*., 2019; Konishi and Ma, 2021; von Wirén *et al*., 2000; Zhang *et al*., 2019), positively regulated ShB resistance (Li *et al*., 2024a; Wu *et al*., 2022) (Figure S2f,i). *TCP19* expression patterns were significantly upregulated under HN conditions (10–15 mM), with a corresponding increase in TCP19 protein levels, which correlated with increased susceptibility to ShB. Conversely, under MN conditions (2–5 mM), both the expression and protein levels of TCP19 were lower (Figures S1b; Figure 2m,n). Elevated *TCP19* expression under HN conditions enhanced susceptibility to ShB, whereas MN conditions were more conducive to controlling ShB. Our study revealed that TCP19 did not act alone in its regulatory role, as evidenced by its interaction with PIL15, which co‐activated *DEP1* while suppressing *NRT1.1B*, thus providing deeper insights into the ShB resistance mechanism (Figure 8j). The observation that *tcp19/NRT1.1BRNAi* and *tcp19/AMT1;2RNAi* plants exhibited longer lesion lengths than WT suggests the involvement of additional TCP19 regulating signalling. This notion is further supported by the enhanced resistance of *tcp19/PIL15RNAi* plants compared to either single mutant, indicating that PIL15 and other potential factors may participate in regulating *NRT1.1B* and *AMT1;2*. These findings highlight the complexity of the regulatory network governing these genes. Both TCP19 and PIL15, members of the bHLH protein family, are essential for regulating plant growth, development, metabolism and responses to environmental stress, suggesting that their interaction affects multiple physiological processes in rice (Pireyre and Burow, 2015). Although HN did not alter PIL15 protein levels, *R*. *solani* infection increased PIL15 protein levels. Interestingly, TCP19 and PIL15 did not exhibit similar regulatory effects on *AMT1;2* (Figure 5m,n), suggesting their involvement in pathways distinct from the ShB resistance mechanism. ChIP‐PCR assays did not detect enrichment of TCP19 and PIL15 to the same promoter regions of downstream gene under MN or HN conditions. This may be attributed to their distinct expression patterns: previous studies have shown that TCP19 is predominantly expressed in roots and stem bases (Liu *et al*., 2021b), while PIL15 is mainly expressed in leaves (Xie *et al*., 2019). The limited spatial overlap between the two proteins may reduce the likelihood of their co‐occupancy within the same cells, making it difficult to capture with ChIP–PCR. Further, the single‐cell transcriptomic approaches may be required to verify this issues in the future. As another interacting protein of TCP19, IDD10 can interact with PIL15 but may not directly regulate *DEP1* and *NRT1.1B*, highlighting the complex regulatory network (Figure 8j). IDD family proteins are crucial for plant growth, development and stress responses (Kumar *et al*., 2019), demonstrating their involvement in various physiological processes when interacting with TCP19 and PIL15. Notably, IDD10 represses *DEP1* potentially by interacting with TCP19 and PIL15 to inhibit the transcriptional activation of *DEP1*. Furthermore, Y2H experiments with truncated forms of TCP19 revealed that IDD10 interacted with the TCP domain at the N‐terminus of TCP19, which is the transcriptionally active domain. This provided evidence for IDD10's role in inhibiting the transcriptional activation ability of TCP19 and PIL15. TCP19 activates *DEP1* expression while inhibiting *AMT1;2* and *NRT1.1B* expression. Interestingly, DEP1 interacted with IDD10, and competitive binding between DEP1 and IDD10 reduced the interaction of IDD10 with TCP19 or PIL15. This reduced the inhibitory effect on DEP1 transcription mediated by TCP19 and PIL15, leading to inhibitory feedback regulation. These data further suggest that IDD10‐mediated inhibition of TCP19‐PIL15 is repressed by a reduction in IDD10 protein levels and an increase in DEP1‐IDD10 complex formation under HN conditions. Thus, TCP19 and PIL15 act as negative regulators of rice ShB resistance, which may cause HN‐induced severity of ShB (Figure 8j). Additionally, the upregulation of *PR1b* in *tcp19* mutants suggests that TCP19 may negatively regulate defence‐related pathways, further reinforcing its role in suppressing ShB resistance (Figure 9k). This highlights a potential link between TCP19‐mediated nitrogen signalling and plant immunity, warranting further investigation.

### Strategies for enhancing disease resistance under high nitrogen fertilization

Given the detrimental effects of high nitrogen on ShB resistance, it is crucial to develop strategies that mitigate these effects while retaining the benefits of increased nitrogen fertilization. To investigate the roles of *tcp19*, *pil15* and *idd10* in yield, we assessed the individual plant yields of *tcp19*, *pil15* and *idd10* mutants. The results showed that the yield per plant in the *tcp19* and *pil15* mutants was slightly higher than that in the wild type, whereas the yield of the *idd10* mutant was comparable to that of the wild type (Figure S7a). Previous studies have established that *NRT1.1B* enhances nitrate uptake efficiency in japonica rice (Hu *et al*., 2015), contributing to improved NUE, with *NRT1.1B* NIL exhibiting a higher grain yield per plant than WT (Hu *et al*., 2015). *AMT1;2* has been shown to improve NUE under nitrogen‐deficient conditions (Lee *et al*., 2020). Although its overexpression does not alter the yield per plant, it increases thousand‐grain weight (Wu *et al*., 2022). Thus, increased expression of *AMT1;2* and *NRT1.1B* may contribute to the observed yield enhancement in the *tcp19* mutant. These findings also provide insights into how *tcp19* may improve NUE beyond DLT‐mediated TRN in rice. NRT1.1B acts as a nitrate sensor and interacts with the phosphate signalling suppressor SPX4 to coordinate the nitrogen–phosphorus utilization (Hu *et al*., 2019). Given that TCP19 regulates *NRT1.1B* and *DEP1* expression, it may serve as a key transcriptional regulator within the nitrogen–phosphorus and carbon–nitrogen signalling networks, orchestrating adaptive responses to fluctuating nutrient availability. Natural variations in *DEP1* have been found to improve NUE, with variants creating a premature stop codon (Huang *et al*., 2022). The *dep1* allele enhances the transcript levels of key ammonium uptake and assimilation genes (e.g. *AMT1;1*, *AMT1;2*, *GS1;2* and *GOGAT1*) and regulates the carbon–nitrogen metabolic balance to affect grain yield and quality (Huang *et al*., 2022; Zhao *et al*., 2019). A mutation in *tcp19* results in reduced *DEP1* expression, but it is unclear whether this reduction contributes to improved NUE. To investigate this, we assessed the expression levels of *AMTs* and *NRT1.1B* in *dep1* mutants. RT‐qPCR results indicated that the expression of *AMTs* and *NRT1.1B* was unaffected by *DEP1* (Figure S7b). These findings suggest that *tcp19* may enhance ShB resistance and improve NUE by regulating both *AMT1;2*‐ and *NRT1.1B*‐mediated N uptake as well as *DEP1*‐mediated N signalling. TCP19‐mediated regulation of *DEP1*, *NRT1.1B* and *AMT1;2* offers a promising strategy for optimizing NUE and ShB resistance in rice cultivation. Our study demonstrated that TCP19 did not directly regulate the genes involved in nitrogen assimilation pathways (Figure S2e). However, under nitrogen deficiency or stress, TCP19 may affect nitrogen metabolism and assimilation pathways. The complexity of the TCP19's response to nitrogen availability highlights the need for further research into the molecular mechanisms underlying its role in nitrogen metabolism regulation. By modulating downstream targets, TCP19 could help allocate nitrogen resources to essential physiological processes while limiting excessive vegetative growth to increase susceptibility to pathogens. Therefore, *TCP19* is a valuable target for breeding disease‐resistant rice varieties.

The findings of this study offer valuable insights for breeding disease‐resistant rice and for managing ShB in rice. It also highlights promising opportunities for improving NUE in rice cultivation. By elucidating the complex regulatory network involving TCP19, PIL15, IDD10, DEP1 and other related genes, future research can deepen our understanding of the molecular mechanisms underlying rice resistance to ShB under various environmental N conditions. Moreover, identifying TCP19, PIL15 and IDD10 as key regulatory factors provides potential targets for genetic manipulation, which could enhance ShB resistance through marker‐assisted selection or gene editing techniques. Overall, this study has significant implications for advancing fundamental knowledge and practical applications in crop improvement and disease management, ultimately contributing to the enhancement of rice production and food security.

## Materials and methods

### Construction of rice mutants and overexpression plants

The ORF sequence of *TCP19* was cloned into a modified pCAMBIA1381 vector, which utilized the maize (*Zea mays*) *ubiquitin* promoter to drive *TCP19* expression. CRISPR/Cas9 genome editing was used to generate mutants and overexpressing plants on the ZH11 background. The seedlings were treated with 0.6% hydrogen peroxide for 20 min, rinsed three times, germinated in sterile water and sown in the substrate soil. The overexpression lines were selected using 50 μg/mL hygromycin B. Mutation sites were confirmed by sequencing. The RNAi silencing vector was constructed using pH7GWIWG2(II).0 (Karimi *et al*., 2005). A 200 bp specific fragment spanning the CDS and containing the 3′UTR of the target gene was selected and cloned into the pH7GWIWG2(II).0 vector by Gateway. Then the vector contained the reverse repeat sequence of the target gene in the T‐DNA region. The transformed strain contained the hygromycin selection marker and was confirmed by qRT‐PCR. The seedlings were cultivated at 28 °C under a 12‐h light/dark cycle in a greenhouse.

### *Rhizoctonia solani* culture and inoculation

The wood veneer was cut into 0.3 cm × 0.7 cm rectangles, sterilized and arranged in a circle on potato dextrose agar (PDA) media. *R. solani* AG1‐IA was incubated at 30 °C for 48–72 h to allow mycelia to grow evenly on the veneer pieces. Rice tillers from 2‐month‐old plants were inoculated with *R. solani*. Mycelium‐covered veneer pieces were placed inside the leaf sheaths, which were then sprayed with sterile water and wrapped with a plastic film to maintain humidity and secure the veneer. Incidence was monitored and photographed every 24 h, and the lesions were recorded approximately 7–10 days after inoculation. Each experiment was repeated at least three times.

### RNA extraction and real‐time quantitative PCR (RT‐qPCR) analysis

Rice leaves were either frozen in liquid nitrogen or stored at −80 °C for RNA extraction. Total RNA was extracted using TRIzol (TaKaRa, Dalian, China), and cDNA was synthesized via reverse transcription using a Vazyme reverse transcription kit (Vazyme, Nanjing, China). *Ubiquitin* served as the internal reference gene, and RT‐qPCR was conducted with Vazyme SYBR ChamQ Universal SYBR qPCR Master Mix (Vazyme). The test employed a two‐step method with a melting curve, and each sample was tested in three technical and three biological replicates. Primers used for RT‐qPCR are listed in Table S3.

### Identification of DEGs under *R. solani* infection and high nitrogen treatment

The pipeline used for pre‐processing and aligning the sequenced raw data from the nitrogen treatment experiment to the reference genome was identical to that described previously (Jiang *et al*., 2023). Following normalization, the gene expression under each of the five nitrogen concentrations was compared with the normal nitrogen conditions to identify differentially expressed genes (DEGs). This analysis was performed separately for the two tissues with a threshold of log_2_ fold change > |1|, *P* <0.05, and adjusted *P* <0.05. DEGs commonly upregulated or downregulated under high nitrogen conditions were selected, and those showing a similar response in both tissues were combined. Genes that responded to *R. solani* infection were identified as candidate DEGs for further analyses. A heatmap was generated using MeV program version 4.9.0.

### MapMan analysis

MapMan version 3.6.0RC1 was used to map the DEGs to various pathways or processes. Regulation and transcription analyses were conducted, and visualizations were created using R Studio version 4.3.2, using the ggplot2 package version 3.5.1. Detailed information on MapMan analysis is provided in Table S3.

### Nitrogen and amino acid content assay in rice

To quantify nitrogen metabolites and amino acids in rice, key compounds involved in nitrogen metabolism, including glutamate (Glu), glutamine (Gln), nitrate (NO_3_^−^) and ammonium (NH_4_^+^), were analysed using specific methods. For Glu, Gln and NO_3_^−^, fresh rice tissues were harvested, immediately frozen in liquid nitrogen and ground into a fine powder using a pre‐chilled mortar and pestle. Tissue extracts were prepared according to the protocols provided in the assay kits (Boxbio, Beijing, China). The absorbance was measured using a spectrophotometer at the wavelengths specified in the manufacturer's instructions. Concentrations were calculated using standard curves generated from known concentrations of Glu, Gln and NO_3_^−^ standards. The NH_4_^+^ content was assessed indirectly by measuring rice root growth following treatment with methylamine. Rice seedlings were cultivated hydroponically under controlled conditions and treated with a nutrient solution containing 3 mM methylamine. Methylamine inhibits ammonium uptake, causing the accumulation of NH_4_^+^ in plant tissues. After 7 days of treatment, root length was measured as an indicator of ammonium accumulation. To quantify the relative impact of ammonium accumulation, root growth inhibition in treated plants was compared to that in control plants grown in a methylamine‐free nutrient solution. All experiments were conducted in biological triplicates. The measured metabolite concentrations and root length data were normalized to fresh tissue weight. Statistical analyses were performed to assess differences among treatments.

### Chromatin immunoprecipitation (ChIP)‐PCR assay

ChIP‐PCR assays were conducted following the procedures described by Je *et al*. (2010) using TCP19‐GFP and PIL15‐GFP transgenic rice plants. Fresh rice leaves were cross‐linked with 1% formaldehyde under vacuum infiltration for 15 min and then quenched with 2 M glycine. Nuclei were isolated from the cross‐linked tissue, and chromatin was sheared to an average fragment size of 200–500 bp by sonication. Sheared chromatin was immunoprecipitated with an anti‐GFP antibody to specifically capture TCP19‐GFP‐DNA and PIL15‐GFP‐DNA complexes. After immunoprecipitation, the protein–DNA cross‐links were reversed by heating at 65 °C overnight, and DNA was purified using phenol–chloroform extraction. Enriched DNA fragments were analysed by PCR using primers specific to the target gene promoter regions. The relative enrichment of DNA fragments in the immunoprecipitated samples compared to the input controls and no‐antibody immunoprecipitation controls was quantified to determine TCP19 binding to the target loci. The PCR primers used for the ChIP assay are listed in Table S3.

### Dual luciferase reporter (DLR) assay

DLR assays were conducted according to previously described procedures (Hellens *et al*., 2005). The *NRT1.1B*, *AMT1;2* and *DEP1* promoters were cloned into the pGreenII 62‐SK vector and transformed into *Agrobacterium tumefaciens* strain GV3101 containing the pSoup plasmid. Equal volumes of *Agrobacterium suspensions*, each containing the transcription factors and promoter constructs, were mixed and infiltrated into the abaxial side of 4‐week‐old *N. benthamiana* leaves using a 1 mL needleless syringe. Following infiltration, the plants were maintained under controlled conditions (24 °C, 16‐h light/8‐h dark cycle) for 48–72 h. Leaf samples were harvested, and luciferase activity was measured using the Dual‐Luciferase Reporter Assay System (Promega, Fitchburg, WI, USA). Primers used for the cloning vectors are listed in Table S3.

### Electrophoretic mobility shift assay (EMSA)

The coding sequences of *TCP19* and *PIL15* were cloned into the pGEX‐5X‐1 vector to produce the GST‐tagged fusion proteins. These constructs were expressed in *Escherichia coli* BL21 (DE3), and protein expression was induced by adding IPTG (0.5 mM) and incubating at 37 °C for 6 h. The GST‐TCP19 and GST‐PIL15 proteins were subsequently purified using GST‐affinity columns (LABLEAD). For probe preparation, synthetic oligonucleotides were biotin‐labelled at their ends and annealed to produce double‐stranded DNA probes. Binding assays were performed using a chemiluminescent EMSA kit (Thermo Fisher Scientific, Waltham, MA, USA). The primer sequences used in the assay are listed in Table S3.

### Yeast one‐hybrid assays

For the yeast one‐hybrid assays (Y1H), promoters of *NRT1.1B*, *AMT1;1*, *AMT1;2*, *AMT1;3* and *DEP1*, containing the TCP19 binding motif, were cloned into the pHISi‐1 vector and transformed into the yeast strain YM4271. The ORF sequences of *TCP19* and *PIL15* were cloned into pGAD424, and the pGAD424‐*TCP19* and pGAD424‐*PIL15* constructs were transformed into yeast cells containing *pHISi‐1*‐*pNRT1.1B*, *pHISi‐1*‐*pAMT1;1*, *pHISi‐1*‐*pAMT1;2*, *pHISi‐1*‐*pAMT1;3* or *pHISi‐1*‐*pDEP1*. Protein–DNA interactions were analysed on SD/‐His media containing various concentrations of 3‐aminotriazole (3‐AT). Primers used for the cloning vectors are listed in Table S3.

### Yeast two‐hybrid assay

For the yeast two‐hybrid assay (Y2H), the ORF sequences of *TCP19* and *PIL15* were cloned into pGAD424 and pGBT9 vectors, *DEP1* was cloned into pGAD424 and *IDD10* was cloned into pGBT9. Additionally, *TCP19* was split into N‐terminal TCP domains (1–240 aa) and C‐terminal regulatory domains (241–387 aa) and cloned into AD and BD vectors, respectively. These fusion vectors were transformed into PJ694A yeast strain using the desired combinations. The yeast transformants were selected using SD/‐Leu‐Trp medium, and diploid yeast cells were transferred to SD/‐Leu‐His‐Trp medium to detect interactions. Primers used for the cloning vectors are listed in Table S3.

### Yeast three‐hybrid assay

For the yeast three‐hybrid assay (Y3H), ORF sequences of *TCP19* and *PIL15* were cloned into pGADT7, *IDD10* was cloned into pBridge MCSI, and *DEP1* was cloned into pBridge MCSII. *TCP19‐pGADT7*, *PIL15‐pGADT7* and *IDD10‐pBridge‐DEP1* were transformed into the Y2HGold yeast strain in desired combinations. Yeast transformants were selected using SD/‐Leu‐Met‐Trp medium, and diploid yeast cells were then transferred to SD/‐Leu‐His‐Trp medium to detect interactions, as well as to SD/‐Leu‐Met/‐His‐Trp medium to assess the effect of the third protein on known interaction pairs. Primers used for the cloning vectors are listed in Table S3.

### BiFC assay

The ORF sequences of *TCP19*, *PIL15* and *IDD10* were amplified and cloned into the BiFC vector, PXCGW (Kim *et al*., 2009). Moreover, *TCP19*, *IDD10* and *DEP1* were cloned into PXNGW to create TCP19(N/C)‐nYFP/cCFP, PIL15‐cCFP and DEP1‐nYFP constructs. These constructs were transformed into *A. tumefaciens* strain GV3101, which were then grown overnight in LB medium with antibiotics, harvested by centrifugation, and resuspended in infiltration buffer (10 mM MgCl_2_, 10 mM MES, pH 5.6 and 150 μM acetosyringone) to an OD_600_ of 0.8. H2B‐RFP was used as a nuclear marker, and P19, a silencing suppressor, was co‐infiltrated. *Agrobacterium* strains containing the interaction pairs were infiltrated into tobacco (*Nicotiana benthamiana*) leaves, and after 36–72 h, fluorescence was observed using an OLYMPUS FV300 laser scanning confocal microscope (Olympus, Tokyo, Japan). Primers used for cloning are listed in Table S3.

### Split firefly luciferase complementation (SFLC) assays

According to a published method (Zhou *et al*., 2018), the ORF sequences of *TCP19*, *PIL15*, *DEP1* and *CIPK31* (negative control) were cloned into the vector pCLUC, and *IDD10* was cloned into pNLUC to create IDD10‐NLUC, TCP19‐CLUC, PIL15‐CLUC, DEP1‐CLUC and CIPK31‐CLUC constructs. These constructs were transformed into *A. tumefaciens* strain GV3101. *A. tumefaciens* strains carrying plasmids encoding the N‐terminal (N‐Luc) and C‐terminal (C‐Luc) fragments of firefly luciferase fused to the proteins of interest were co‐infiltrated into the leaves of 4‐week‐old *N. benthamiana* plants. Bacterial cultures were grown overnight in LB medium with appropriate antibiotics, harvested by centrifugation and resuspended in infiltration buffer (10 mM MgCl_2_, 10 mM MES, pH 5.6 and 150 μM acetosyringone) to an OD_600_ of 0.8. Equal volumes of *Agrobacterium* suspensions containing N‐Luc and C‐Luc constructs were mixed and infiltrated into the abaxial side of tobacco leaves using a 1 mL needleless syringe. The plants were maintained under controlled conditions (24 °C, 16‐h light/8‐h dark cycle) for 48–72 h post‐infiltration. Luciferase activity was assessed by spraying the infiltrated leaves with 1 mM luciferin substrate and measuring bioluminescence using a CCD camera. IDD10 and CIPK31, known non‐interacting proteins (Li *et al*., 2024b), were used as negative controls, whereas RbohD and BIK1, which are known interacting proteins (Zhou *et al*., 2018), served as positive controls. The interaction strength was quantified by analysing the luminescence intensity. Primers used for the cloning vectors are listed in Table S3.

### Co‐immunoprecipitation (co‐IP) and western blot (WB) assay

The ORF sequences of *TCP19*, *IDD10* and *DEP1* were cloned into the pGD3GGm vector, whereas those of *PIL15* and *TCP19* were cloned into the pGD3G3Flag vector to generate the constructs. These constructs were transformed into the *A. tumefaciens* strain GV3101. Each interaction pair was mixed with P19 at a 3:5:5 ratio and used for infiltration into tobacco leaves. Forty‐eight hours after infiltration, 1 g of leaf sample was collected, and total protein was extracted using an extraction buffer consisting of 150 mM NaCl, 2.5 mM Tris–HCl, 1 mM EDTA, 10% glycerol, 0.1% NP40, 2% PVPP, 10 mM DTT and a protease inhibitor cocktail. The total proteins were then immunoprecipitated using the anti‐GFP or anti‐FLAG magnetic beads (Sigma Aldrich, St. Louis, MO, USA).

Protein samples were boiled with 5× loading buffer and analysed by western blotting using anti‐GFP, anti‐MYC and anti‐FLAG antibodies (ABmart, Shanghai, China). Signal detection was performed using a Tanon 4600 system (Tanon, Shanghai, China), and the relative band densities were calculated using ImageJ software. Primers used for cloning are listed in Table S3.

### Statistical analysis

All collected data were processed using Prism 8 software (GraphPad Software) and expressed as the mean ± standard error. The statistical significance between two groups was analysed using Student's *t*‐test, while multiple group comparisons were assessed by one‐way ANOVA. Differences among the samples were considered significant at *p <* 0.05.

## Conflict of interest

The authors declare no competing interests.

## Author contributions

YHX planned and designed the study. TGZ, HC, QJL and HYZ performed experiments. TGZ, HC and XJ analysed the data. TGZ, YHX, XJ, KHJ and XFZ wrote the manuscript. All authors approved the final manuscript.

## Supporting information


**Figure S1** Analysis of the TCP gene family in rice.
**Figure S2** TCP19 downstream gene screening and validation.
**Figure S3** Analysis of TCP19 in nitrogen uptake and metabolism, and its regulatory role in downstream targets.
**Figure S4** Structural analysis of TCP19 and identification of PIL15 mutants and overexpression lines.
**Figure S5** Analysis of the regulatory mechanism of IDD10 in downstream genes.
**Figure S6** Investigation of interactions between DEP1, IDD10 and TCP19/PIL15 proteins.
**Figure S7** Analysis of gene‐related yield and nitrogen.

**Table S1** Common regulatory genes under *R. solani* infection and high‐nitrogen treatment.
**Table S2** Detailed information of MapMan analysis.
**Table S3** Primers (5′‐3′) used in this study.

## Data Availability

The data that supports the findings of this study are available in the supplementary material of this article.
