# Supplementary material for: TCP19 regulates nitrogen‐dependent sheath blight susceptibility by modulating nitrogen uptake and signalling in rice
Source: Plant Biotechnol J. 2025 Jun 26;23(9):4140–57. doi: 10.1111/pbi.70224 (PMC12392934; doi:10.1111/pbi.70224)
Supplement: Supplementary file 1 — Figure S1 Analysis of the TCP gene family in rice. Figure S2 TCP19 downstream gene screening and validation. Figure S3 Analysis of TCP19 in nitrogen uptake and metabolism, and its regulatory role in downstream targets. Figure S4 Structural analysis of TCP19 and identification of PIL15 mutants and overexpression lines. Figure S5 Analysis of the regulatory mechanism of IDD10 in downstream genes. Figure S6 Investigation of interactions between DEP1, IDD10 and TCP19/PIL15 proteins. Figure S7 Analysis of gene‐related yield and nitrogen. [file PBI-23-4140-s002.docx]

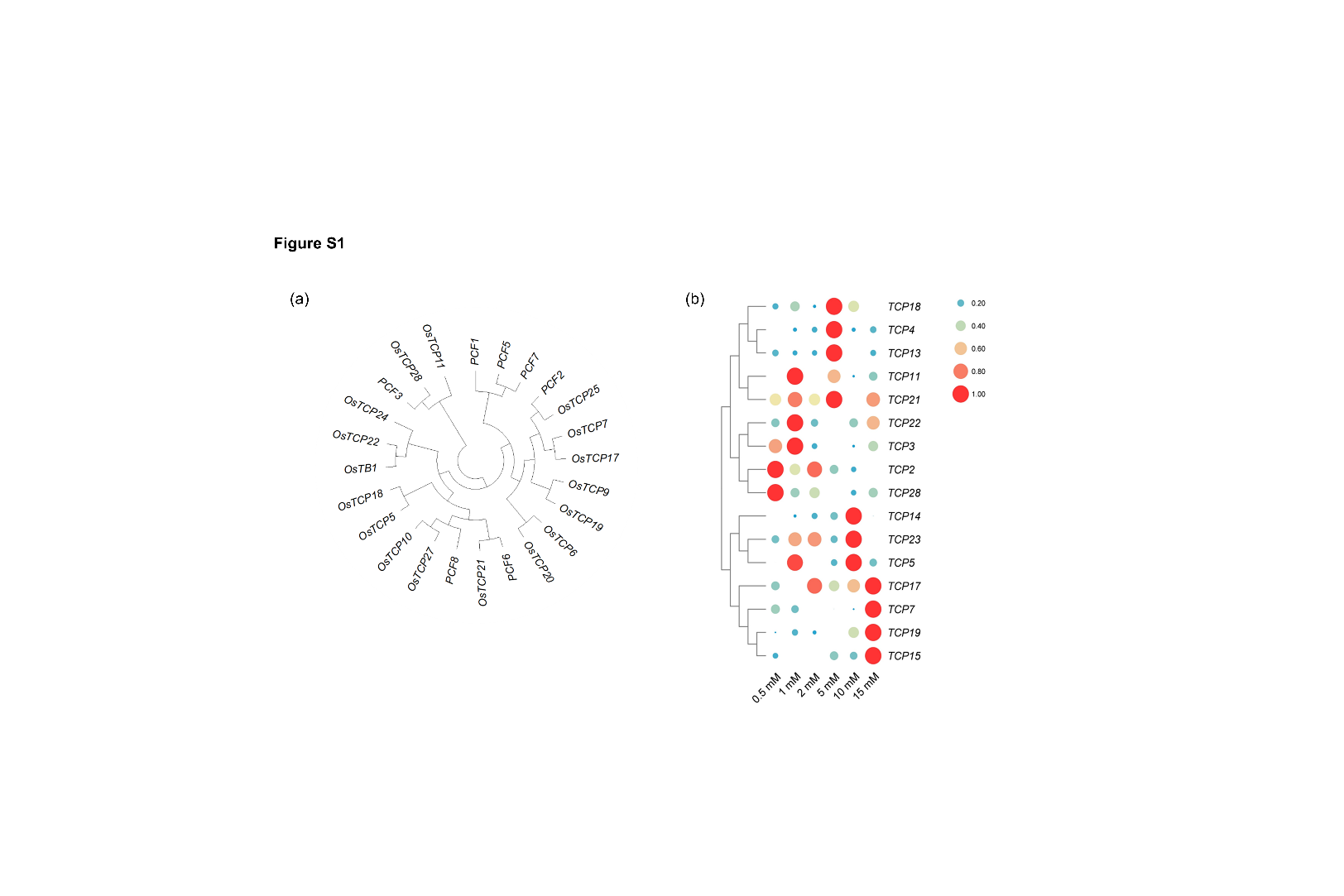


**Supplementary Figure 1.** Analysis of the TCP gene family in rice. **(a).** Evolutionary analysis of the TCP gene family in rice. **(b).** Heatmap of *TCP* family gene expression treatment using nitrogen.


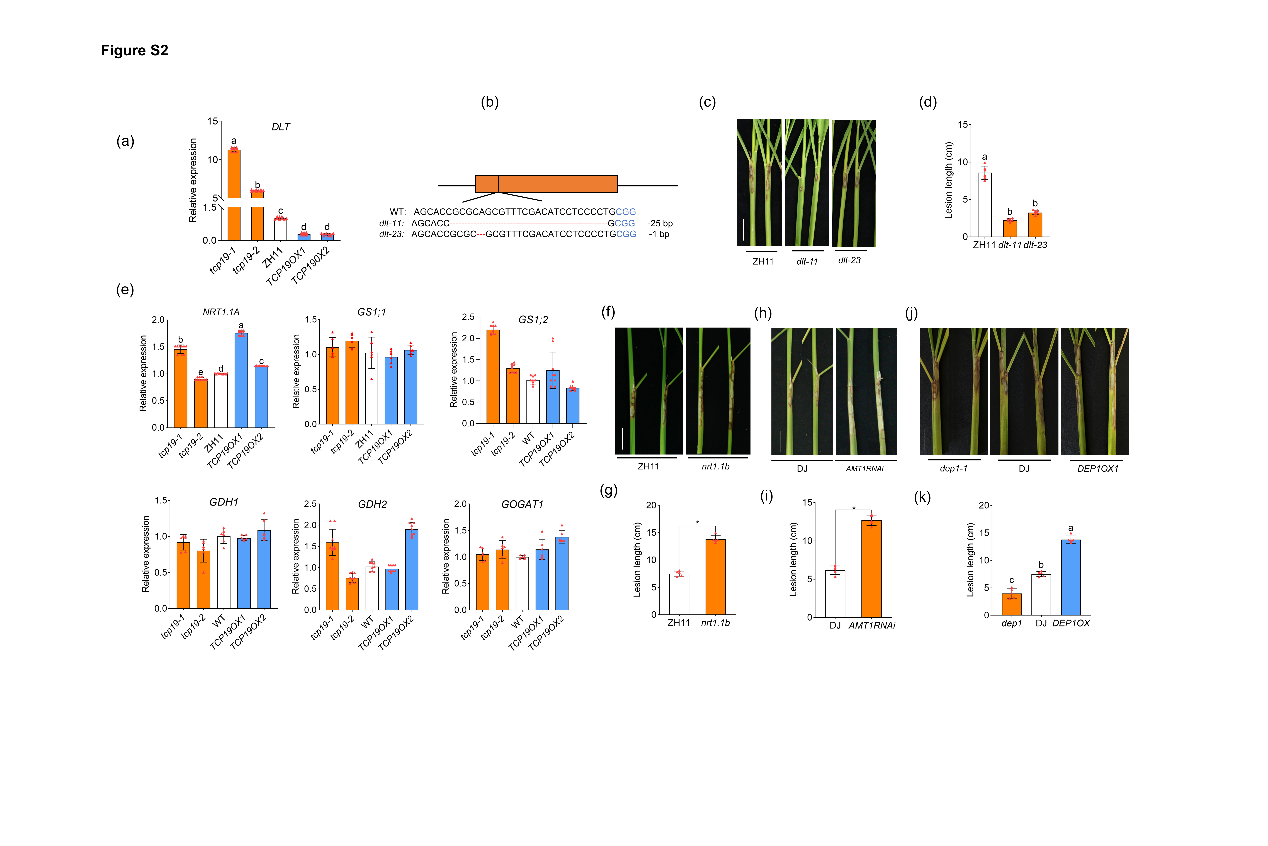


**Supplementary Figure 2.** TCP19 downstream gene screening and validation. **(a).** *DLT* expression in *tcp19* mutants, *TCP19 OXs*, and WT. **(b).** High-throughput sequencing identification of *dlt* CRISPR/Cas9 gene-edited mutants. Compared with WT plants, mutant sequencing analysis showed 25 bp and 1 bp knockout at the exon, leading to a frameshift. **(c, d).** Identification of the incidence after *R. solani* inoculation and statistical analysis of disease spot length in *dlt* mutants and WT. One-way ANOVA was used to analyze significant differences between groups. Different lowercase letters above the bars indicate significant differences (*P < 0.05*). At least six replicates were used for each assay. Scale bar = 2 cm. **(e).** Expression levels of *NRT1.1A,* *GS1;1*, *GS1;2*, *GDH1*, *GDH2*, and *GOGAT1* in *tcp19* mutants, *TCP19OXs*, and WT. **(f–k).** Assessment of disease incidence and statistical analysis of lesion length after *R. solani* inoculation in the *nrt1.1b*, *AMT1RNAi*, *dep1*, and *DEP1 OX* lines and their respective WT. Significant differences between groups were determined using t-tests or one-way ANOVA. Asterisks or different lowercase letters indicate significant differences (*P < 0.05*). At least six replicates were used for each assay. Scale bars = 2 cm.

15
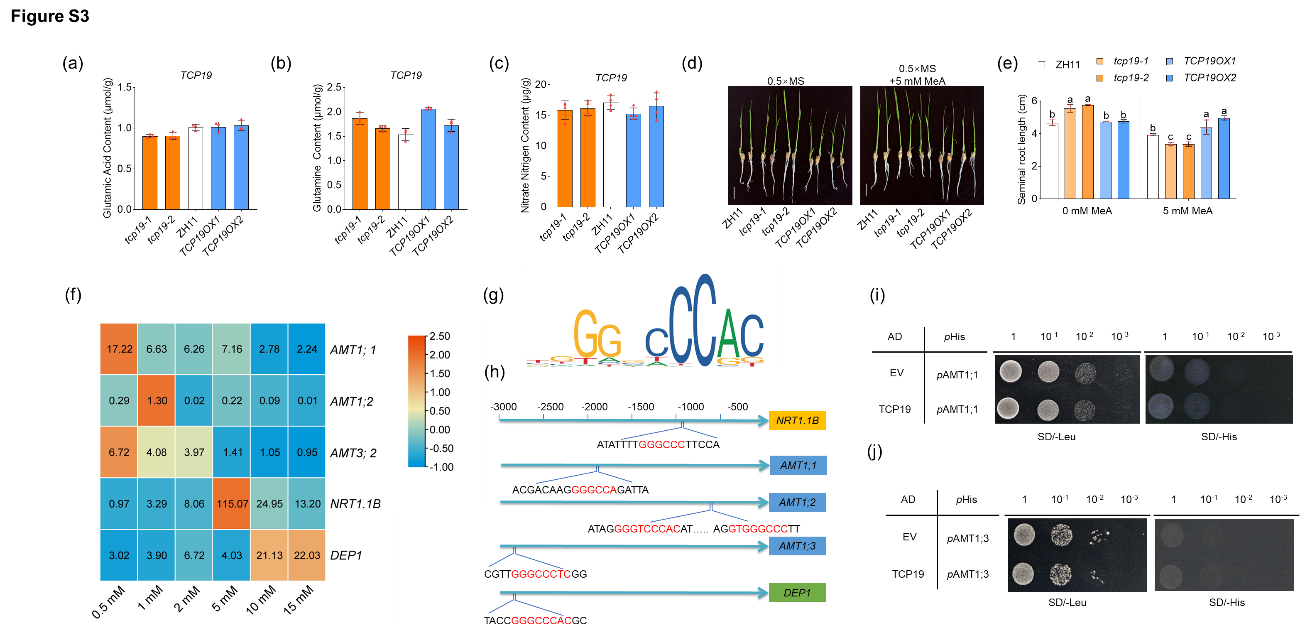


**Supplementary Figure 3.** Analysis of TCP19 in nitrogen uptake and metabolism, and its regulatory role in downstream targets. **(a–c).** Levels of glutamate, glutamine, and nitrate in *tcp19* mutants, *TCP19 OXs*, and WT. Significant differences between groups were analyzed using one-way ANOVA. Different lowercase letters above the bars indicate significant differences (P < 0.05). At least three replicates were used for each assay. **(d, e).** Phenotypic observations and root length measurements in *tcp19* mutants, *TCP19 OXs*, and WT following methylammonium (MeA) treatment. Significant differences between groups were analyzed using one-way ANOVA. Different lowercase letters above the bars indicate significant differences (P < 0.05). At least three replicates were used for each assay. Scale bar = 2 cm. **(f).** Heatmap of *AMT1s*, *NRT1.1B*, and *DEP1* expression treatment using nitrogen. **(g).** Rice consensus DNA motif bound by the TCP19 protein. **(h).** Predicted TCP19 binding motifs in the promoters of *NRT1.1B*, *AMT1;1*, *AMT1;2*, *AMT1;3*, and *DEP1*. **(i, j).** Y1H assay results showing no interaction between TCP19 and the promoters of *AMT1;1* and *AMT1;3*.


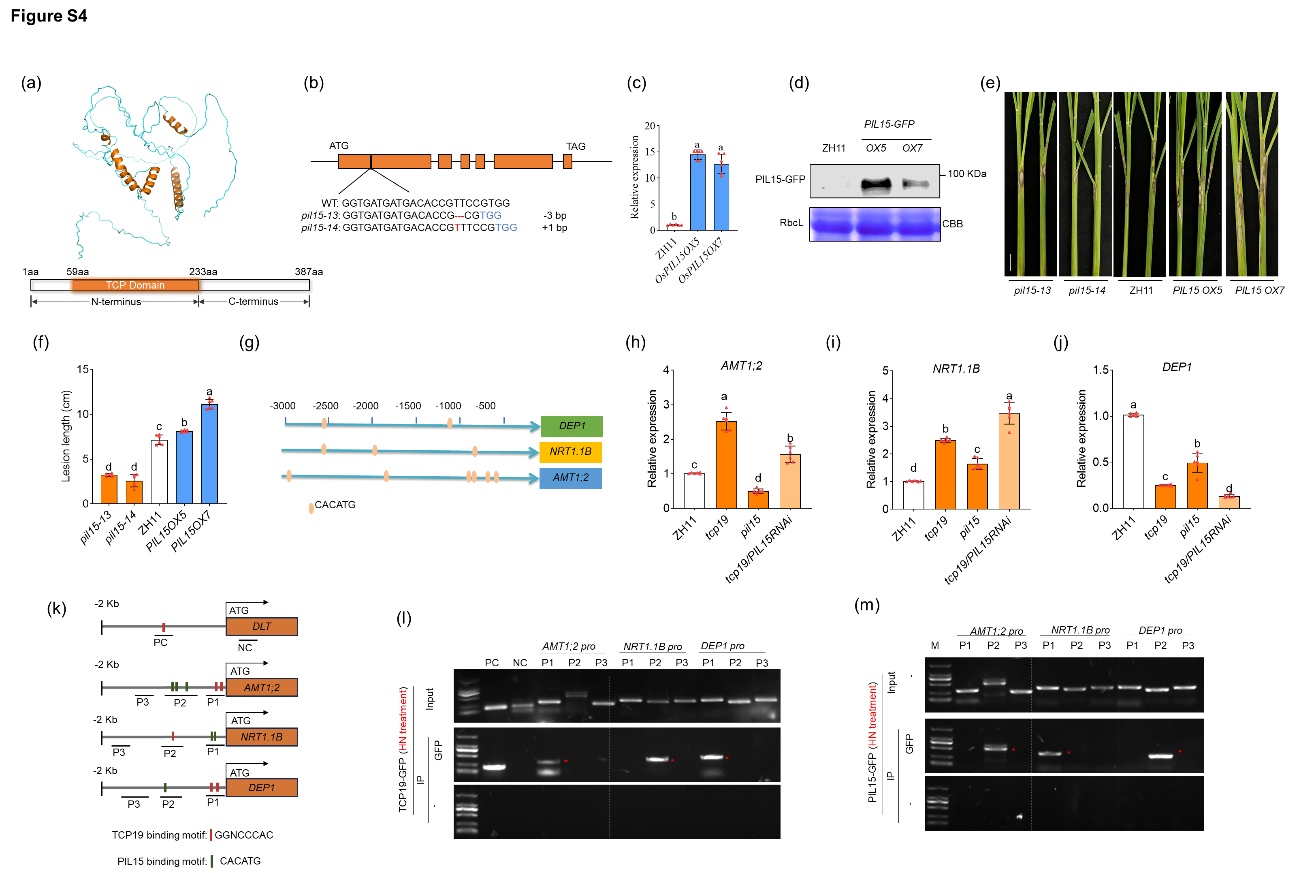


**Supplementary Figure 4.** Structural analysis of TCP19 and identification of PIL15 mutants and overexpression lines. **(a).** TCP19 protein structure and localization of the TCP domain in TCP19 protein. **(b).** High-throughput sequencing identification of *pil15* CRISPR/Cas9 gene-edited mutants. Compared with WT plants, mutant sequencing analysis showed a 3 bp knockout and a 1 bp insertion in the first exon. **(c, d).** RT-qPCR and WB analysis of *PIL15* expression in overexpressed lines and WT. Significant differences between groups in the RT-qPCR assay were analyzed using one-way ANOVA. Different lowercase letters above the bars indicate significant differences (P < 0.05). At least six replicates were used for each assay. **(e, f).** Phenotypic observations and statistical analysis of disease incidence after *R*. *solani* inoculation in *pil15* mutants, *PIL15 OXs*, and WT. Significant differences between groups were analyzed using one-way ANOVA. Different lowercase letters above the bars indicate significant differences (*P < 0.05*). At least six replicates were used for each assay. Scale bar = 2 cm. **(g).** PIL15 binding motif in the promoters of *NRT1.1B*, *AMT1;2*, and *DEP1*. **(h–j).** Expression levels of *AMT1;2*, *NRT1.1B* and *DEP1* in *tcp19*, *pil15*, *tcp19/PIL15 RNAi* and WT. Significant differences between groups were analyzed using one-way ANOVA. Different lowercase letters above the bars indicate significant differences (*P < 0.05*). At least three replicates were used for each assay. **(k–m).** ChIP–PCR analyses of *AMT1;2*, *NRT1.1B*, and *DEP1* promoter regions in *TCP19-GFP OX* and *PIL15-GFP OX* under HN treatment. Red asterisks indicate specific DNA bands.


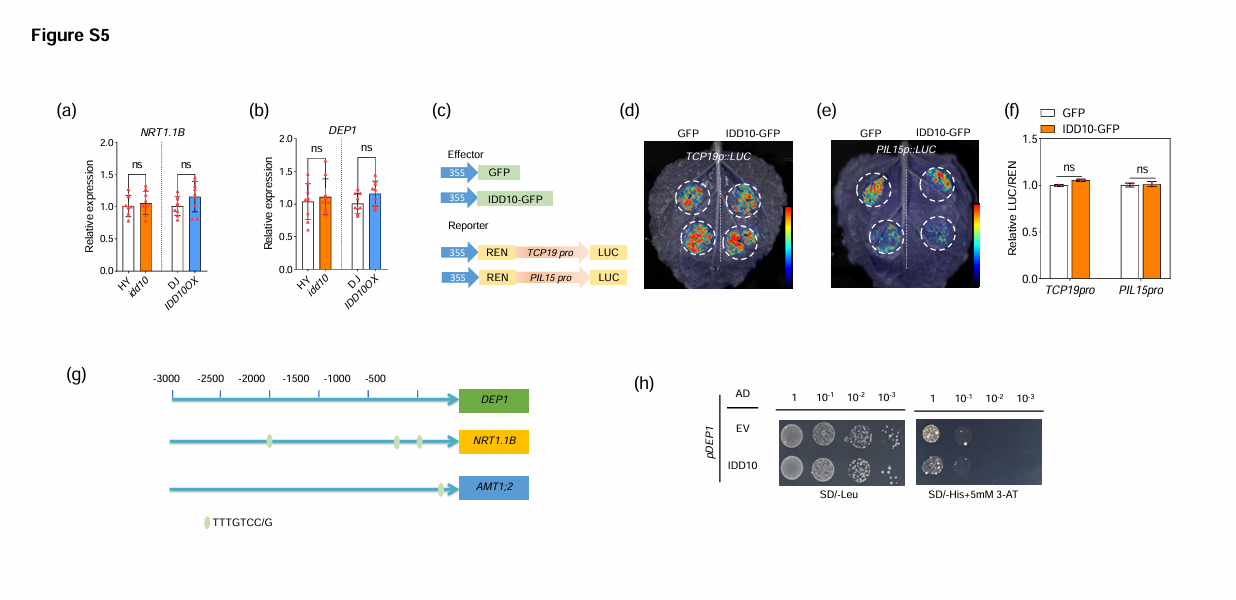


**Supplementary Figure 5.** Analysis of the regulatory mechanism of IDD10 in downstream genes. (**a, b).** Expression levels of *NRT1.1B* and *DEP1* in *idd10* mutants, *IDD10 OX*, and WT. Significant differences between groups were analyzed using t-tests. ns indicates no significant differences (*P ≥ 0.05*). At least six replicates were used for each assay. **(c–f).** LUC reporter assays showed that IDD10 did not regulate the promoters of *TCP19* and *PIL15*. Luciferase activities were shown relative to those obtained after transfection with 35S::GFP and the reporter construct, which was set to 1. Significant differences between groups were analyzed using a t-test, and ns indicates no significant differences (*P ≥ 0.05*). At least three replicates were used for each assay. **(g).** IDD10 binding motifs in the promoters of *NRT1.1B* and *AMT1;2* but not in *DEP1*. **(h).** Y1H assay results showed that IDD10 did not interact with the promoter of *DEP1*.


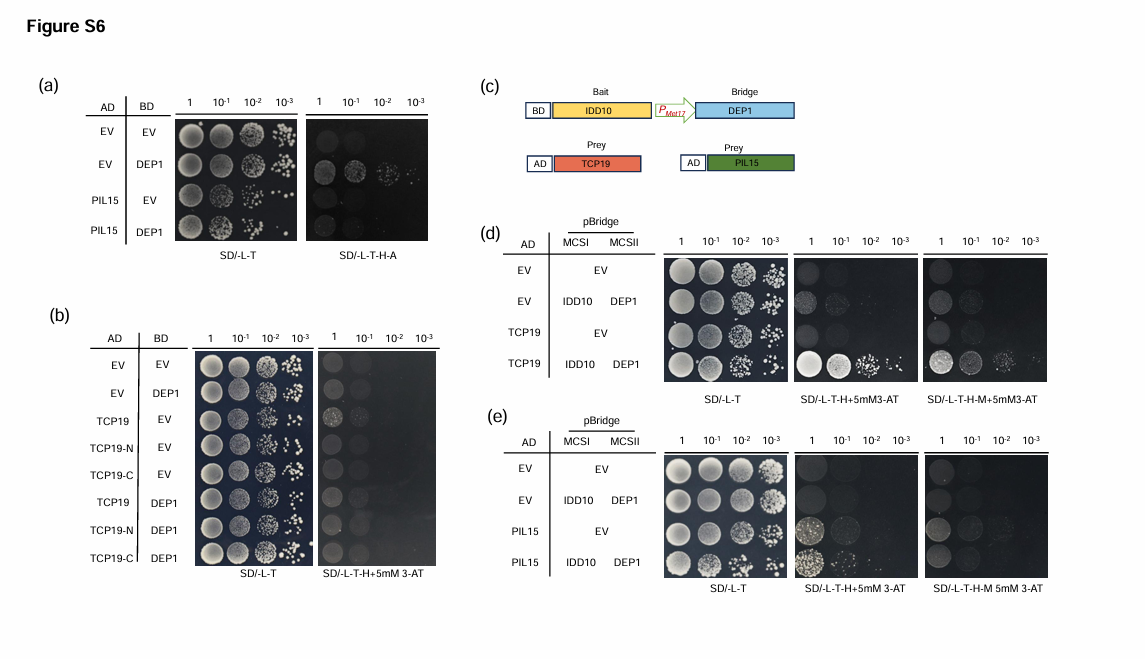


**Supplementary Figure 6.** Investigation of interactions between DEP1, IDD10, and TCP19/PIL15 proteins. **(a, b).** Y2H assay results showed that PIL15 and TCP19 did not interact with DEP1. **(c–e).** Y3H assays revealed interactions between IDD10 and TCP19 or PIL15 both in the presence and absence of DEP1.


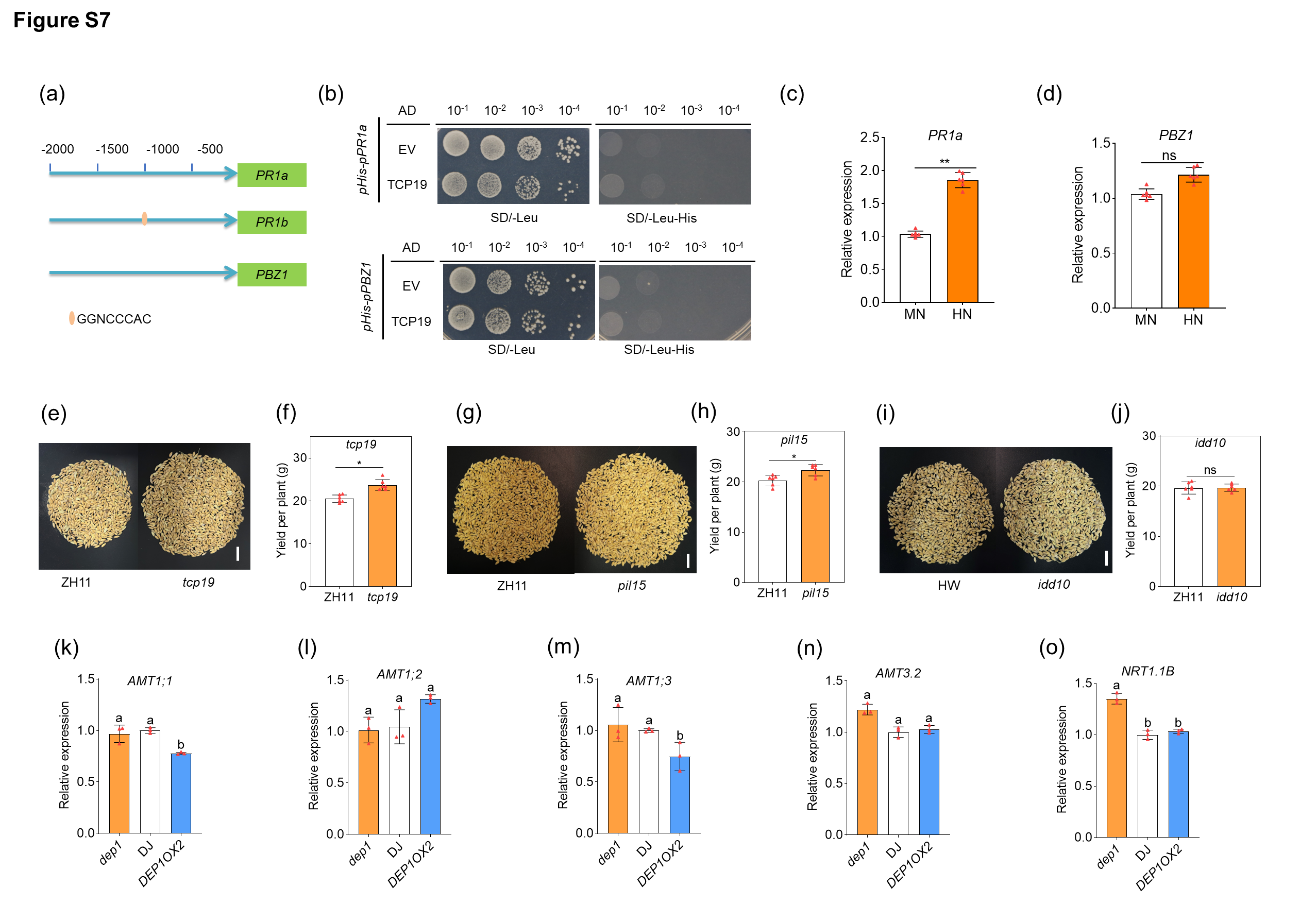


**Supplementary Figure 7.** Analysis of gene-related yield and expression. **(a).** TCP19 binding motif in the promoter of *PR1b* but not in *PR1a* and *PBZ1*. **(b).** Y1H assay results showed that TCP19 did not interact with the promoter of *PR1a* and *PBZ1*. **(c, d).** Expression levels of *PR1a* and *PBZ1* under MN and HN conditions. One-way ANOVA was used to analyze significant differences between groups. Different lowercase letters above the bars indicate significant differences (*P < 0.05*). At least six replicates were used for each assay. **(e–j).** Yield per plant of *tcp19*, *pil15*, *idd10*, and WT. Significant differences between groups were analyzed using t-tests. Asterisks indicate significant differences (*P < 0.05*), and ns indicates no significant differences (*P≥0.05*). Scale bar = 2 cm. **(k-o).** Expression levels of *NRT1.1B,* *AMT1;1*, *AMT1;2*, *AMT1;3*, *AMT3.2*, and *NRT1.1B* in *dep1* mutant, *DEP1OX*, and WT. Statistical analysis was conducted to assess differences in disease spot lengths between groups. One-way ANOVA was used to analyze significant differences between groups. Different lowercase letters above the bars indicate significant differences (*P < 0.05*). At least six replicates were used for each assay.
